# D801N in *ATP1A3*-encoded Na/K-ATPase alpha 3 causes cardiac arrhythmogenesis through sodium-calcium exchanger–mediated calcium overload

**DOI:** 10.1172/jci.insight.197721

**Published:** 2026-04-08

**Authors:** Minu-Tshyeto K. Bidzimou, Padmapriya Muralidharan, Zhushan Zhang, Danyal Raza, Daniel Needs, Bo Sun, Robin M. Perelli, Mary E. Moya-Mendez, P.K. Rakesh Manivannan, Arsen S. Hunanyan, Abbigail Helfer, Christine Q. Simmons, Alfred L. George, Donald M. Bers, Nenad Bursac, Mohamad A. Mikati, Andrew P. Landstrom

**Affiliations:** 1 Department of Cell Biology and; 2 Department of Pediatrics, Division of Cardiology, Duke University School of Medicine, Durham, North Carolina, USA.; 3 Department of Biomedical Engineering and; 4 Division of Pediatric Neurology and Developmental Medicine, Department of Pediatrics, Duke University, Durham, North Carolina, USA.; 5 Department of Pharmacology, Northwestern University Feinberg School of Medicine, Chicago, Illinois, USA.; 6 Department of Pharmacology, University of California Davis, Davis, California, USA.; 7 Department of Neurobiology, Duke University, Durham, North Carolina, USA.

**Keywords:** Cardiology, Cell biology, Neuroscience, Arrhythmias, Cardiovascular disease, Ion channels

## Abstract

Short QT syndrome is a heritable arrhythmia disorder linked to sudden cardiac death. We recently identified that individuals with alternating hemiplegia of childhood (AHC), a rare neurodevelopmental disorder, can exhibit shortened corrected QT intervals and elevated risk for ventricular fibrillation. This is especially true for patients with AHC heterozygous for the recurrent ATP1A3-D801N variant, though the underlying cardiac mechanism remains unclear. We hypothesized that the D801N missense impairs Na^+^/K^+^-ATPase function, causing Ca^2+^ overload, shortened action potential duration (APD), and arrhythmias. Using in silico modeling and patient-derived induced pluripotent stem cell cardiomyocytes (iPSC-CMs^D801N^), we observed shorter APD, elevated intracellular and sarcoplasmic reticulum Ca^2+^ levels, and delayed afterdepolarizations (DADs) compared with WT. Additionally, increased Ca²^+^ influx via the Na^+^/Ca^2+^ exchanger (NCX1) during depolarization was observed in iPSC-CMs^D801N^. Simulations and in vitro experiments suggest that reduced ATPase function accelerated inactivation of L-type Ca^2+^ channels. Pharmacologic inhibition of NCX1 with ORM-10103 normalized APD and reduced DADs. These findings support a Ca^2+^-mediated mechanism for arrhythmogenesis in ATP1A3-D801N carriers and identify NCX1 as a potential therapeutic target.

## Abstract 2

The mechanistic link between the alternating hemiplegia of childhood-associated ATP1A3-D801N mutation and cardiac short QT and ventricular arrhythmia risk

## Introduction

Short QT syndrome (SQTS) is a rare heritable disorder characterized by a shortened QTc interval on a cardiac electrocardiogram (ECG) (1). Individuals with SQTS are at a greater risk of ventricular arrhythmias and sudden cardiac death (SCD) (2, 3). More than 90% of patients with SQTS have an unknown genetic cause and uncertain cellular mechanisms, thus limiting treatment, screening, and prevention (4–6). This lack of mechanistic understanding is reflected in the absence of pharmacologic therapies to treat the disease (7–10).

In light of the unclear molecular underpinnings of SQTS, attention has turned toward fundamental ion transport mechanisms, which are critical for maintaining cardiac excitability and may contribute to arrhythmogenic processes. Na^+^/K^+^ ATPases (NKA) belong to a class of proteins that pump intracellular Na^+^ out of the cell and extracellular K^+^ into the cell, against their respective electrochemical gradients to maintain the ionic gradients needed for generating action potentials and other physiological phenomena (11). This maintenance of myocyte homeostasis promotes the normal function of other ion-transporting proteins, including the Na^+^/Ca^2+^ exchanger (NCX1), a membrane protein that facilitates transport of Ca^2+^ ions out of the cell with the use of the Na^+^ gradient (11). By limiting intracellular Na^+^ accumulation, NKA preserves NCX1 efflux during the action potential plateau and repolarization, thereby preventing excessive Ca^2+^ loading that would otherwise alter CDI of inward current and the balance of repolarizing currents. In this way, NKA indirectly shapes action potential duration by stabilizing both Ca^2+^ handling and the timing of the electrogenic NCX1 current (11, 12). The activity of NKA also contributes to an appropriate resting membrane potential (RMP) of cardiomyocytes, which is crucial for action potential dynamics (13). One NKA isoform, *ATP1A3*-encoded alpha 3 (ATP1A3), has an undefined role in the heart. Classically, variants in *ATP1A3* are known to cause a variety of neurologic diseases, with alternating hemiplegia of childhood (AHC) being the most prominent. AHC presents in infancy with episodic hemiplegia, autonomic dysfunction, dystonia, seizures, and oculomotor abnormalities (14). AHC is also associated with increased mortality, with an etiology that has been attributed to sudden unexplained death in epilepsy (SUDEP) (15, 16). Recently, pathogenic variants in *ATP1A3* have been found to be associated with short QTc on ECG and predisposition to ventricular arrhythmias (17, 18). Specifically, ATP1A3-D801N is the most recurrent missense accounting for 30%–40% of cases among genotype-positive individuals with AHC, and it is associated with shortened QTc (19). Moreover, patients heterozygous for this variant are at an increased risk of life-threatening ventricular arrhythmias (17, 18). This raises the possibility that abnormal repolarization of the cardiac myocytes due to the ATP1A3-D801N variant may represent a novel mechanism of SQTS and SCD.

In this study, we investigated the expression and function of ATP1A3 in the heart and how the D801N variant may lead to abnormal repolarization and proarrhythmia. We found that diminished Na^+^/K^+^-ATPase activity associated with ATP1A3-D801N promotes NCX1-mediated Ca^2+^ influx at depolarized potentials, which shortens APD, increases intracellular Ca^2+^ levels and sarcoplasmic reticulum (SR) Ca^2+^ stores, and consequently predisposes to delayed afterdepolarizations (DADs). We also identified NCX modulation as an approach to rectify APD and decrease DAD prevalence.

## Results

### ATP1A3-D801N has lower NKA function.

**Figure 1:**
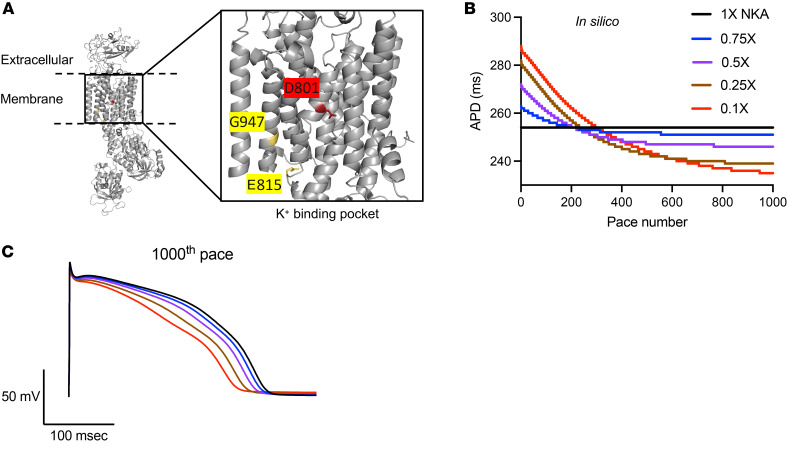
ATP1A3-D801N decreases NKA function and is predicted to decrease cardiomyocyte depolarization time. (**A**) Variant mapping of the 3 most QTc penetrant missenses among patients with AHC. Crystal structure of the human ATP1A3 is shown with the D801, E815, and G947 residues highlighted. Residues in yellow have 10%–20% penetrance of SQTc while residues in red have a penetrance higher than 50% (D801N, 68.57%; E815K, 19.05%; G947R, 12.50%). (**B** and **C**) In silico ToR-ORd model of human cardiomyocyte predicting APD as a function of pace number under different NKA functional states.

To begin investigating the role of missense variants in *ATP1A3* and short QT development, we first mapped the location of the 3 most common variants in ATP1A3 within the protein structure. We have previously found that the D801N, E815K, and the G947R variants in ATP1A3 are associated with short QT on ECG (18). We mapped these 3 variants to the atomic-level structure of human ATP1A3 and categorized them based on their degree of penetrance, with D801N having high penetrance for short QT. We found that all 3 variants localize to the K^+^ binding pocket of ATP1A3 (Figure 1A). Since previous evidence has shown that the D801N variant lacks significant ATPase activity (20), we next tested the functional effect of this variant in vitro. HEK293T cells natively express ATP1A3 (21), and overexpression of ATP1A — as well as exposure to ouabain, a selective inhibitor of ATP1A3 — are both toxic to these cells (22). While overexpression of WT ATP1A3 caused cell death, we found that overexpression of ATP1A3-D801N had normal survival. In the presence of ouabain, cell survival was rescued in the setting of overexpression of a ATP1A3 mutant that renders it ouabain resistant; however, ouabain resistant ATP1A3-D801N failed to rescue survival (Supplemental Figure 1, more details in the Supplemental Results; supplemental material available online with this article; https://doi.org/10.1172/jci.insight.197721DS1). These findings suggest that D801N strongly impairs ATP1A3 function.

Using established transcriptional data, we found that *ATP1A3* comprises 26.3% of the total *ATP1A* transcript levels in healthy human myocardium (Supplemental Figures 2 and 3). Conversely, RNA-seq conducted in 3-day-old (P3) and 3-month-old mice found that *Atp1a3* transcripts contribute less than 1% of *Atp1a* in WT murine hearts (Supplemental Figure 4). We found a similar low relative abundance of *Atp1a3* among other in vivo experimental models. *Atp1a3* transcripts contribute 2% of total *Atp1a* in rat and sheep hearts (Supplemental Figures 5 and 6), *atp1a3* is not expressed in zebrafish heart, and there are no orthologs of *ATP1A3* in either *Caenorhabditis elegans* or *drosophila melanogaster* (Supplemental Results). These findings suggest that human myocardium, unlike commonly used experimental animal models, expresses substantial ATP1A3 and may be uniquely susceptible to the functional effect of the D801N variant. We next employed an in silico model of human cardiomyocyte electrophysiology, the Tomek-Rodriguez (ToR-ORd) model, to evaluate the effect of decreased NKA function (23). In line with our previous findings suggesting that the D801N variant shortens the QT interval and APD (18), we found that reducing NKA function (by 25%) was associated with shorter APD (Figure 1, B and C). This model also predicted more depolarized RMP as expected for reduced NKA function (Supplemental Figure 7). Overall, these results suggest that loss of proper ATP1A3 function in human myocardium is associated with APD shortening and RMP depolarization.

### ATP1A3-D801N is associated with faster cardiac myocyte repolarization and DADs.

**Figure 2:**
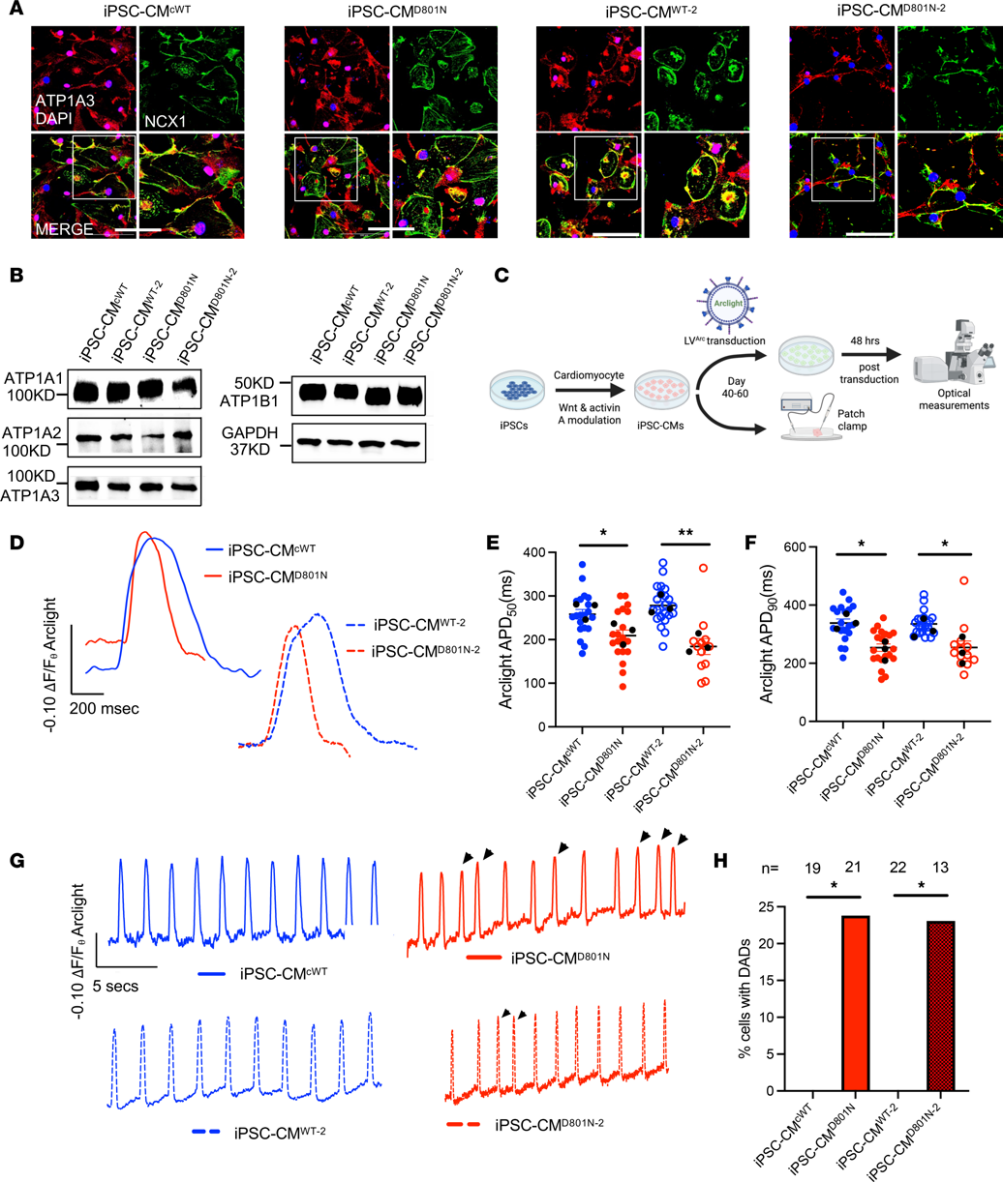
iPSC-CM^D801N^ have shortened APD and delayed afterdepolarizations. (**A**) Representative images of iPSC-CMs immunofluorescence: ATP1A3 (green), NCX1 (red), and DAPI (blue). (**B**) Western blots of ATP1A1-3, ATP1B1, and GAPDH expression in iPSC-CMs^WT^ and iPSC-CMs^D801N^. (**C**) Schematic of experimental approach for iPSC-CMs using whole cell patch clamp or a genetically encoded fluorescent voltage sensor (Arclight), packaged in lentivirus (LV). (**D**) Representative fluorescence tracing of Arclight. (**E** and **F**) Fluorescence change duration at 50% and 90% from baseline (representing APD_50_ and APD_90_, respectively). For iPSC-CM^cWT, iPSC-CM^D801N, iPSC-CM^WT-2, iPSC-CM^D801N-2, *n* = 19, 21, 22, and 13, respectively. (**G**) Representative tracings of iPSC-CMs spontaneous oscillations in Arclight transduced iPSC-CMs. In spontaneously fluorescing cells, arrows identify irregularly triggered fluorescence indicative of delayed afterdepolarizations (DADs). (**H**) Bar graph quantifying percent cells with DADs. Live cell experiments were statistically analyzed with a hierarchical approach. Fischer’s exact test was conducted on **H**. **E** and **F** were statistically tested with a hierarchical approach.*P < 0.05, **P < 0.01.

To determine the mechanism of ATP1A3-D801N–mediated alterations in cardiac repolarization and arrhythmogenesis, we leveraged 2 iPSC lines from D801N^+^ AHC probands with short QTc (male iPSC^D801N^ and female iPSC^D801N-2^) (18). Each patient-derived line was compared with a corresponding control: an isogenic WT was used as a control for the male iPSC^D801N^ line and an unrelated healthy male WT line for the female iPSC^D801N-2^ (iPSC^cWT^ and iPSC^WT-2^, respectively) (18). Using immunofluorescence, we found that expression of ATP1A3 colocalized with the NCX1 in both iPSC-CMs^WT^ and in iPSC-CMs^D801N^ (Figure 2A and Supplemental Figure 8). Expression of ATP1A1, ATP1A2, ATP1A3, and ATP1B1 in iPSC-CMs were confirmed by Western blot in iPSC-CMs^WT^ and iPSC-CMs^D801N^ (Figure 2B). We recently demonstrated that iPSC-CMs^D801N^ exhibit a shortened APD, depolarized RMP, and DADs by single-cell patch clamp recording (18). We employed a genetically encoded fluorescence voltage sensor (Arclight) to evaluate cellular membrane depolarizations (Figure 2C). Live cell imaging of Arclight transduced iPSC-CMs revealed significantly shorter repolarization time and the presence of DADs in CMs differentiated from both D801N lines compared with respective controls (Figure 2, D–H, and Supplemental Figure 9). Application of ouabain to iPSC-CM^WT-2^ was associated with shortening of APD_50_, APD_90_, and more depolarized maximum diastolic potential (MDP) compared with vehicle treatment, all of which were similar to action potential kinetics of iPSC-CMs^D801N^ (Supplemental Figure 10) (18). Together, these findings demonstrate that D801N does not alter expression or trafficking of ATP1A3 to the membrane, yet it is associated with shortened APD and DAD generation.

### iPSC-CM^D801N^ have Ca^2+^ miniwaves, greater SR Ca^2+^ store levels, and higher cytosolic Ca^2+^ levels.

**Figure 3:**
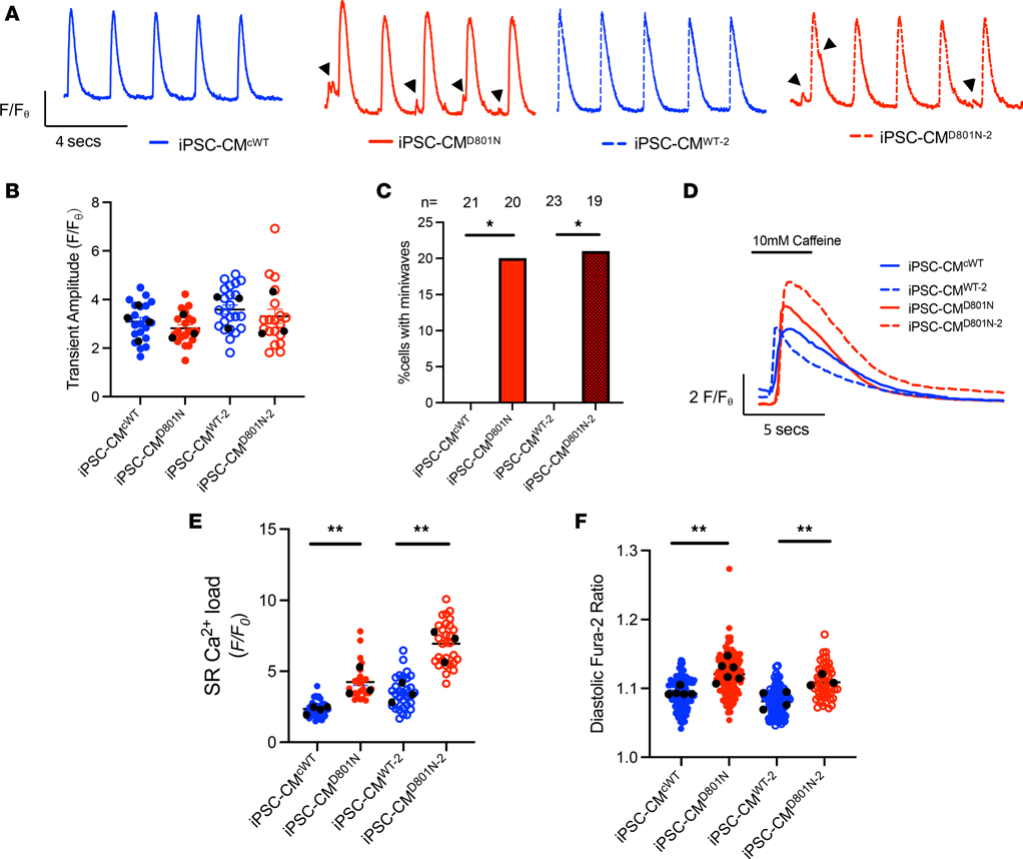
iPSC-CM^D801N^ have miniwaves, higher SR [Ca^2+^] store content, and higher [Ca^2+^]_i_. (**A**) Representative Ca^2+^ transients of Cal-520 loaded iPSC-CMs^WT^ and iPSC-CMs^D801N^ paced at 0.5 Hz. Black arrows indicate miniwaves. (**B**) Quantification of transient fluorescence amplitude. (**C**) Bar graph demonstrating percent cells with miniwaves. Fisher’s exact test. (**D**) Representative trace of Cal-520 fluorescence upon application of 10 mM caffeine as a measure of sarcoplasmic reticulum (SR) Ca^2+^ load. (**E**) SR store fluorescence amplitude. For iPSC-CM^cWT, iPSC-CM^D801N, iPSC-CM^WT-2, iPSC-CM^D801N-2, *n* = 43, 27, 31, and 29, respectively. (**F**) Diastolic fura-2 ratio after 0.5Hz pacing. For iPSC-CM^cWT, iPSC-CM^D801N, iPSC-CM^WT-2, iPSC-CM^D801N-2, *n* = 133, 156, 87, and 53, respectively. Black dots represent experimental means. Live cell experiments were statistically analyzed with a nested approach. Fischer’s exact test was conducted on **C**. *P < 0.05, **P < 0.01.

Ion transport coupling between outward NKA and inward NCX1 currents allow the cell to repolarize after each depolarization, and changes in NKA function and [Na^+^]_i_ can affect NCX1-mediated Ca^2+^ flux (13). To explore the influence of NKA on Ca^2+^ dynamics in our cellular model, we conducted live cell imaging of Cal-520–loaded iPSC-CMs. We observed that iPSC-CMs^D801N^ have similar Ca^2+^ transient amplitudes and similar transient resolution time (tau) when compared with iPSC-CMs^WT^ (Figure 3, A–C, and Supplemental Figure 11). We also found that iPSC-CMs^D801N^ are predisposed to the generation of miniwaves, which manifest as small, truncated Ca^2+^ transients, and are a known cellular mechanism of triggered arrhythmias (24). Conversely, iPSC-CMs^WT^ demonstrated no miniwaves (Figure 3C and Supplemental Figure 12). We next measured SR Ca^2+^ store levels with the application of 10 mM caffeine and found higher SR Ca^2+^ store levels in iPSC-CMs^D801N^ compared with iPSC-CMs^WT^ (Figure 3, D and E). Using Fura-2 with prepacing of cardiomyocytes at 0.5 Hz, we found higher basal levels of [Ca^2+^]_i_ in iPSC-CMs^D801N^ compared with controls (Figure 3F), which was in close agreement with our in silico model showing that lower NKA function were associated with higher [Ca^2+^]_i_ and Ca^2+^ stores (Supplemental Figure 13).

To determine whether elevated SR Ca^2+^ content was associated with greater Ca^2+^ leak from the SR, we explored RyR2-mediated leak by measuring Ca^2+^ sparks with confocal live cell imaging. We found that iPSC-CMs^D801N^ had comparable spark frequency compared with controls (Supplemental Figure 14, A and B). Furthermore, while sparks from iPSC-CMs^D801N^ cells had higher amplitude, all other Ca^2+^ spark parameters, such as spark width and duration, were similar to iPSC-CMs^WT^ (Supplemental Figure 14, C–E). The higher Ca^2+^ sparks amplitude would tend to promote propagating Ca^2+^ waves and limit the rise in SR Ca^2+^ content. When normalized to SR Ca^2+^ store levels, which is positively associated with SR Ca^2+^ leak levels, we found that iPSC-CMs^D801N^ had similar normalized Ca^2+^ leak compared with control cells (Supplemental Figure 14, F and G). This suggests that the prevalence of miniwaves in D801N myocytes is due to increased SR Ca^2+^ content rather than a fundamental alteration in RyR2 gating. Overall, we found that iPSC-CMs^D801N^ have higher levels of Ca^2+^ within the cytosol, as well as the SR, and are predisposed to Ca^2+^ miniwaves when compared with iPSC-CMs^WT^.

### iPSC-CM^D801N^ exhibit greater NCX1 Ca^2+^ influx at positive membrane potentials.

**Figure 4:**
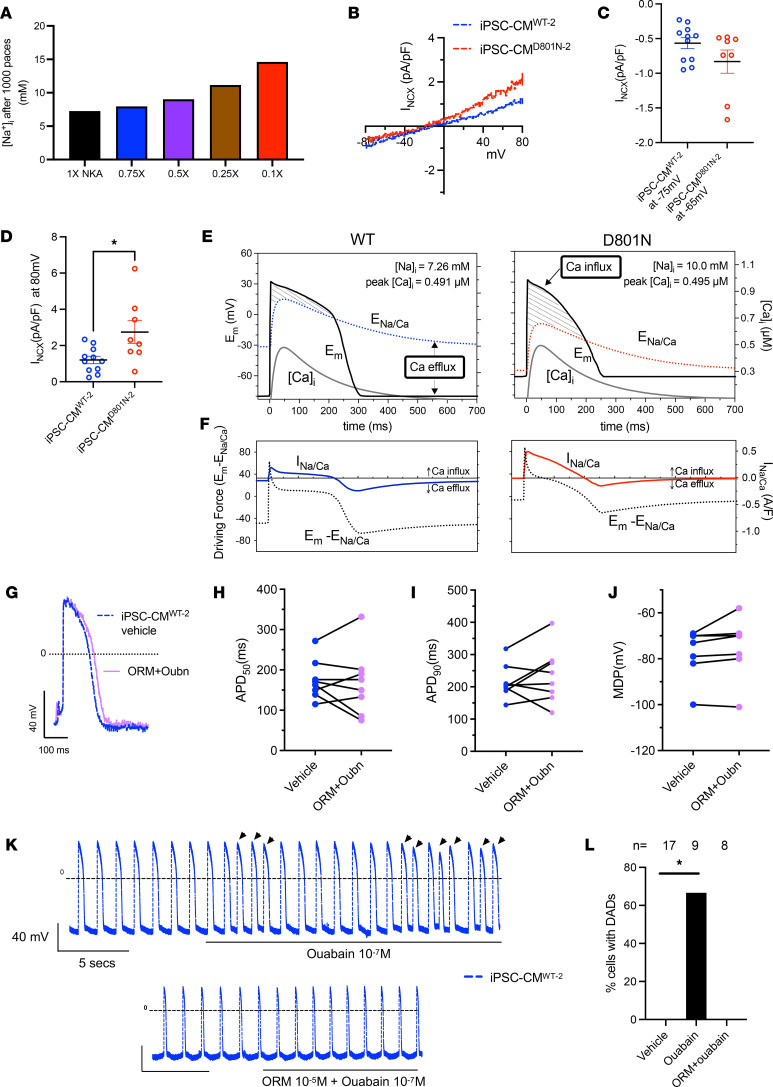
iPSC-CM^D801N^ have greater NCX1 Ca^2+^ influx at positive membrane potential. (**A**) In silico ToR-ORd human cardiomyocyte model; graph illustrates [Na^+^]_i_ as a function of NKA functional states after 1,000 paces. (**B**) IV curve of I_NCX_ in iPSC-CM^WT-2^ and iPSC-CM^D801N-2^. (**C**) I_NCX_ current of iPSC-CM^WT-2^ and iPSC-CM^D801N-2^ at their respective maximum diastolic potential. (**D**) I_NCX_ current of iPSC-CM^WT-2^ and iPSC-CM^D801N-2^ at +80mV. (**E** and **F**) Graphs illustrating changes in E_Na/Ca_ during the action potential of WT and D801N cardiomyocytes. NCX mediated Ca^2+^ influx is thermodynamically favored when E_m_ > E_Na/Ca_ and NCX-mediated Ca^2+^ efflux is favored when E_m_ < E_Na/Ca_. (**G**) AP trace of iPSC-CM^WT-2^ treated with vehicle and with 1 × 10^–5^ M ORM10103 + 1 × 10^–7^ M ouabain. (**H**–**J**) Action potential duration at 50% repolarization (APD_50_), 90% repolarization (APD_90_), and maximum diastolic potential (MDP) of vehicle treated and ouabain + ORM10103–treated iPSC-CM^WT-2^. (**K** and **L**) Ouabain-mediated DAD rescue by cotreatment with ORM-10103. Arrows indicate delayed afterdepolarizations (DAD) events). Results were statistically analyzed with a Mann-Whitney *U* test or Wilcoxon matched-pairs test. Fischer’s exact test was conducted on K. *P < 0.05, **P < 0.01.

We further investigated the link between ATP1A3-D801N and Ca^2+^ overload. Given our findings of increased [Ca^2+^]_i_ (likely driven by a rise in [Na^+^]_i_), we hypothesized that NCX1 function is altered, thus loading iPSC-CMs^D801N^ with intracellular Ca^2+^ in the setting of a reduced [Na^+^] gradient. In silico simulations show how lower NKA function increases [Na^+^]_i_ (Figure 4A) and Ca^2+^ influx (Supplemental Figure 15). We calculated NCX1 Ca^2+^ efflux using the tau decay of Ca^2+^ extrusion after maximal SR store Ca^2+^ release and found that iPSC-CM^D801N^ had slowed Ca^2+^ efflux than iPSC-CM^cWT^ (Supplemental Figure 16 and 17). That is consistent with the elevated [Na^+^]_i_ despite the elevated [Ca^2+^]_i_ release (Figure 3, D and E). SERCA activity during [Ca^2+^]_i_ decline was higher in iPSC-CM^D801N^ cells compared with control (Supplemental Figure 18), which reflects the higher intracellular and SR Ca^2+^ levels and an increase in SERCA versus NCX dominance in cytosolic Ca^2+^ removal SERCA. The iPSC-CMs^D801N^ had comparable NCX1 expression with iPSC-CMs^WT^ by Western blot (Supplemental Figure 19), suggesting that the altered NCX function is due to the altered conditions, rather than NCX1 protein expression level. To further test this finding in vitro, we measured NCX1 current as a function of membrane potential under more controlled [Ca^2+^]_i_ and [Na^+^]_i_ conditions. In iPSC-CM^D801N-2^, inward I_NCX_ (Ca^2+^ efflux) was similar to iPSC-CMs^WT^ when cells were held at negative membrane potentials (18). Conversely, outward I_NCX_ at positive membrane potentials was higher in iPSC-CM^D801N-2^ compared with iPSC-CM^WT-2^, reflecting greater Ca^2+^ influx during depolarized membrane potentials (Figure 4, B–D). Although the Ca^2+^ flux toward new steady state can be contributed by other compensatory mechanisms, we wanted to evaluate the NCX1 specific contribution. To accomplish this, we calculated the driving force of NCX1 flux in D801N and WT cardiomyocytes (25, 26) and found higher Ca influx in D801N (Figure 4, E and F).

To test whether Ca^2+^ influx can be modulated to be a net efflux and consequently alter APD, we used ORM-10103, a small molecule that preferentially inhibits NCX1 Ca^2+^ influx (27). After pretreatment with ouabain to partially inhibit NKA (and raise [Na^+^]_i_), treatment of iPSC-CM^WT-2^ with ORM-10103 normalized the APD and MDP comparable with vehicle-treated iPSC-CM^WT-2^ parameters (Figure 4, G–J). We also observed that ORM-10103 prevented ouabain-induced DADs in iPSC-CM^WT-2^ (Figure 4, K and L). Taken together, we conclude that ATP1A3-D801N promotes NCX1-mediated Ca^2+^ influx at positive membrane potentials, limiting net NCX Ca^2+^ efflux, and that inhibition of NCX1-mediated Ca^2+^ influx normalizes APD shortening associated with ATP1A3 inhibition and prevents DAD generation.

### Lower NKA function promotes faster L-type Ca^2+^ channel inactivation.

**Figure 5:**
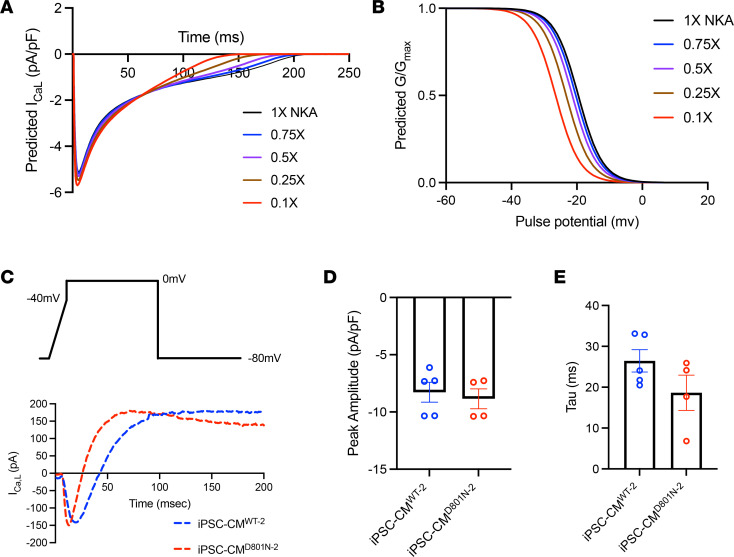
Lower NKA functional states lead to faster L-type Ca^2+^ channel inactivation. (**A** and **B**) In silico TOr-ORd human cardiomyocyte model using decreasing NKA functional states after 1,000 paces. Peak LTCC current (**A**) and calculated LTCC steady state inactivation (**B**). (**C**–**E**) Patch clamp recordings. (**C**) Current density raw trace of iPSC-CM^WT-2^ and iPSC-CM^D801N-2^ using a 1-step activation protocol. (**D**) Bar graph illustrating current density per cell. (**E**) Bar graph illustrating tau of decay per cell. A Mann-Whitney *U* test was conducted, and no statistical difference was found.

To determine the biophysical mechanism of shortened APD, we hypothesized that phase 2 of the action potential is shorter in iPSC-CMs^D801N^ because of Ca^2+^-dependent inactivation (CDI) of the L-type Ca^2+^ channel (LTCC). In silico simulations suggest that lower NKA function was associated with a very slight increase in peak I_Ca,L_, accelerated inactivation and a negative shift in steady state LTCC availability (Figure 5, A and B). Additionally, we found that lower NKA activity was associated with faster I_Kr_ inactivation but with lower peak current, suggesting a compensatory response to shortened APD rather than the driving effect (Supplemental Figure 20). In vitro voltage*-*clamp of iPSC-CM^D801N-2^ had a trend toward faster inactivation compared with control, with similar peak current amplitudes (Figure 5, C–E). However, this interpretation is complicated by the residual outward currents present in our relatively physiological solutions. Thus, our ATP1A3-D801N cardiomyocytes may have faster I_Ca,L_ inactivation.

### Inhibiting NCX1 Ca^2+^ influx with ORM-10103 rescues APD and prevents DADs in iPSC-CM^D801N^.

**Figure 6:**
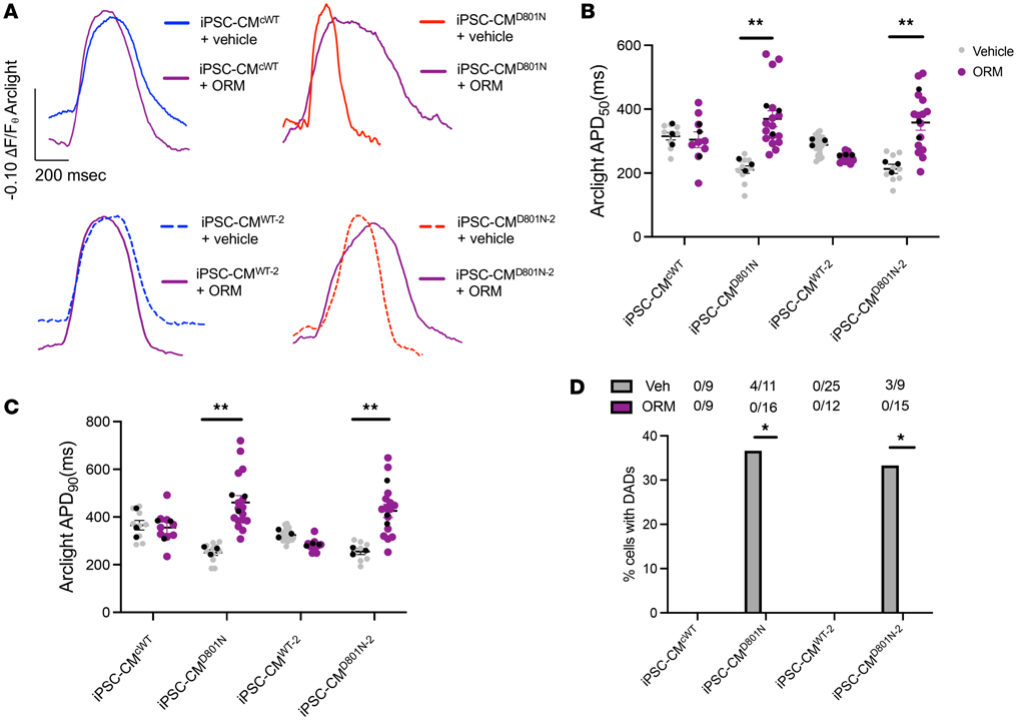
NCX1 Ca^2+^ influx inhibition with ORM-10103 rescues APD and prevents DADs in iPSC-CMs^D801N^. Rescue of APD and DADs with ORM-10103 on Arclight transduced cardiomyocytes. (**A**) Representative fluorescence tracing of vehicle and ORM-10103–treated cells. (**B** and **C**) Fluorescence change duration at 50% and 90% from baseline representing APD_50_ and APD_90_, respectively. (**D**) Percent cells with DADs when treated with vehicle or 1 × 10^–5^ M ORM-10103. Black dots represent experimental means. Results were statistically analyzed with a hierarchical test. Fischer’s exact test was conducted on **D**. Panels B and C were statistically analyzed with a hierarchical test. *P < 0.05, **P < 0.01.

**Figure 7:**
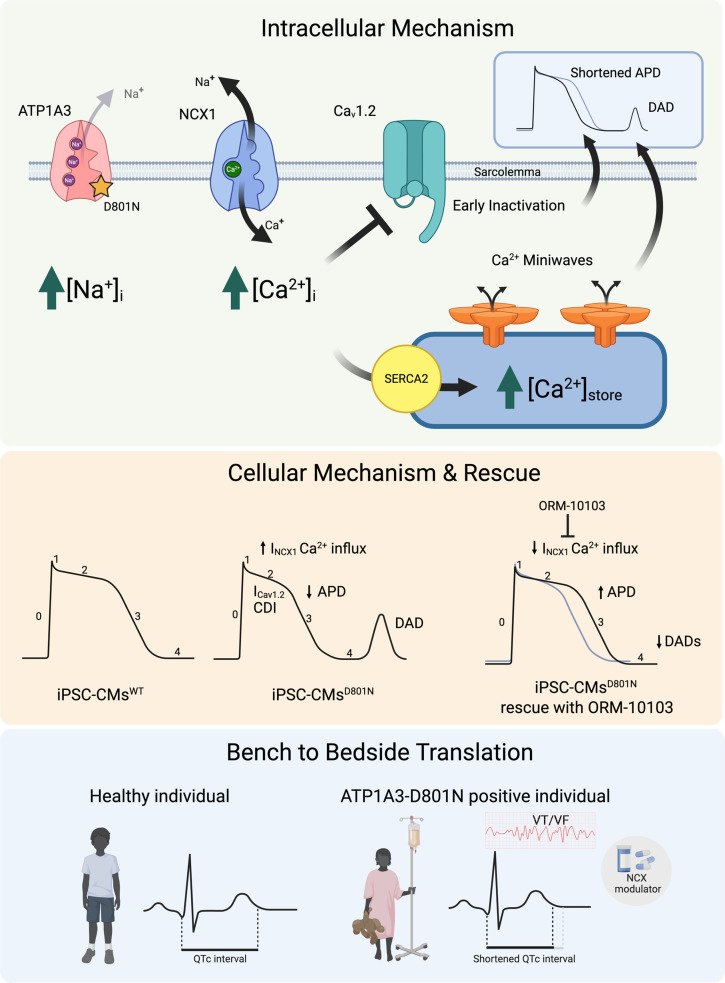
Cellular mechanism of ATP1A3-D801N. Expression of ATP1A3-D801N leads to a decrease in NCX1 mediated Ca^2+^ efflux. This causes a buildup of intracellular [Ca^2+^]_i_ and SR store, favoring miniwaves and delayed afterdepolarizations (DADs). In the setting of Ca^2+^ overload, Ca_v_1.2 rapidly inactivates which shortens phase 2 and leads to a shorter action potential (APD). Inhibition of NCX1-mediated Ca^2+^ influx with ORM-10103 rescues both APD and DADs in iPSC-CMs^D801N^, suggesting that NCX1 can be modulated in patients with ATP1A3-D801N to increase ventricular repolarization time and decrease arrhythmic risk.

We tested the feasibility of rescuing the shortened APD and DAD generation in iPSC-CMs^D801N^ using outward-biased I_NCX_ inhibition. Using Arclight-transduced cardiac myocytes, we measured APD_50_ and APD_90_ in vehicle-treated and ORM-10103–treated cardiomyocytes. We found that ORM-10103 increased APD_50_ and APD_90_ in iPSC-CMs^D801N^ but had no effect on APD in iPSC-CMs^WT^ (Figure 6, A–C). This is consistent with the idea that there is relatively little outward I_NCX_ at normal [Na^+^]_i_ levels (13). We also observed that ORM-10103 suppressed DADs in iPSC-CMs^D801N^ (Figure 6D). In summary, we found that inhibition of NCX1-mediated Ca^2+^ influx with ORM-10103 rescued APD and prevented DADs in the D801N iPSC-CM models, which suggests a potentially new therapeutic approach to shortened QTc in ATP1A3-D801N (Figure 7).

## Discussion

Our findings presented herein demonstrate that individuals with ATP1A3-D801N have a myocardium that is susceptible to arrhythmia. In our experiments using AHC patient–derived iPSC-CMs^D801N^ and the ToR-ORd model, we found that ATP1A3-D801N leads to elevation of [Na^+^]_i_, which limits NCX1-mediated Ca^2+^ efflux and also increases Ca^2+^ influx at depolarized potentials. Over time, this leads to Ca^2+^ overload, both in the cytoplasm and SR. These Ca^2+^ overloaded cells contribute to the APD and DAD phenotype via outward INCX and miniwaves, respectively.

The modulators of cardiac myocyte repolarization are still emerging. More than 90% of patients with SQTS have an unknown genetic cause and unknown cellular mechanism, thus limiting treatment, screening, and prevention of life-threatening ventricular arrhythmias (5, 6). Challenges in identifying mechanisms of SQTS have slowed the development of models that can recapitulate disease and test potential treatments. Most genotype-positive individuals with heritable SQTS have gain-of-function variants in K^+^-channel–encoding genes such as *KCNH2* and *KCNQ1* (28). These variants lead to increased outward current, which shorten phase 3 of the ventricular action potential and consequently shorten its APD and shortened QT intervals on ECG. Conversely, loss-of function variants in *CACNA1C* encoded LTCC have been associated with SQTS (29). It has been shown in vitro that SQTS-associated *CACNA1C* variants exhibit smaller inward current that contributes to shorter phase 2 of the action potential (29). To date, there is no mechanism to our knowledge to explain how variants in NKA genes cause faster cardiac repolarization or arrhythmias, although reduced NKA function is expected to reduce the outward Na-pump current and shift INCX in the outward direction, thus shortening APD. Multiple clinical studies have been reported in the treatment of SQTS, and hydroquinidine has been shown to be the most successful in increasing APD and reducing arrhythmic burden (7). However, those studies overly represented K^+^ channel variants. While targeting NCX1 activity may represent a rational strategy to mitigate Ca^2+^ overload secondary to ATP1A3-D801N–mediated NKA dysfunction, such an approach is likely context dependent. In SQTS unrelated to NKA variants, or under conditions of limited Ca^2+^ loading, NCX1 inhibition could impair contractility and alter electrophysiological stability, underscoring the need for careful therapeutic evaluation.

NKA function has historically been a target for pharmacologic intervention of heart failure with cardiac glycosides. Partial inhibition of NKA increases [Na^+^]_i_ and promotes NCX1-dependent Ca^2+^ loading of the cardiac myocytes and improved contractility (30). Of note, significant inhibition of ATP1A3 with the cardiac glycoside ouabain has been shown to decrease NKA function and lead to pathologic increases in [Ca^2+^]_i_ and DAD-triggered arrhythmias (31). We find evidence of both elevated [Ca^2+^]_i_ and DADs in the setting of the D801N missense variant, as well as higher [Na^+^]_i_ with lower NKA function in the ToR-ORd model. While we are the first to show this in the cardiac myocyte, previous reports have found that D801N affects K^+^ binding to NKA, which impairs the pump cycling from the high energy state phosphorylated conformation (E2P) to the unphosphorylated conformation (E2) and decreases NKA pump function. (20). We find that ouabain causes shortened APD, and we observe both shortened APD and Ca^2+^ overload phenomenon in iPSC-CMs^D801N^, suggesting that lower ATP1A3 function leads to increased [Na^+^]_i_ and [Ca^2+^]_i_, which promote generation of Ca^2+^ miniwaves and DADs in the myocardium.

Excess intracellular Ca^2+^ shortens APD by altering the balance of inward and outward ionic currents through both direct Ca^2+^-dependent modulation and secondary changes in electrogenic Ca^2+^ handling. Elevated cytosolic Ca^2+^ accelerates CDI of LTCCs, reducing inward I_Ca,L_ during the plateau phase and thereby favoring earlier repolarization (32). In parallel, Ca^2+^ overload suppresses I_K1_ and can enhance repolarizing K^+^ currents such as I_Kr_, shifting the net current balance toward APD shortening and increased susceptibility to arrhythmogenesis (33). Experimental and computational studies further demonstrate that Na^+^ overload–driven Ca^2+^ accumulation via NCX influx augments repolarizing I_NaK_ and I_NaCa_, contributing to APD shortening despite increased Ca^2+^ influx and Ca^2+^ transient amplitude (34).

Ca^2+^-activated K^+^ currents also participate in Ca^2+^-mediated APD modulation. Small-conductance Ca^2+^-activated K^+^ (SK) channels (I_SK_) can enhance repolarization and shorten APD when activated by low-to-moderate increases in intracellular Ca^2+^ (35). However, I_SK_ exhibits a biphasic dependence on [Ca^2+^]_i_, such that more severe Ca^2+^ overload induces Ca^2+^-dependent block, limiting its contribution during the plateau phase (36). Accordingly, in pathological settings characterized by augmented Ca^2+^ influx such as in ATP1A3-D801N–expressing ventricular myocytes, the contribution of I_SK_ to phase-2 APD shortening is anticipated to be reduced, with APD shortening dominated instead by enhanced CDI of I_Ca,L_ and Ca^2+^-driven changes in other repolarizing and electrogenic transport currents.

Consistent with this integrated framework, intact-heart experiments and human ventricular modeling demonstrate that elevations in cytosolic and SR Ca^2+^, particularly at higher pacing rates, enhance CDI of I_Ca,L_, modify RyR2 gating, and act synergistically with rate-dependent increases in repolarizing K^+^ currents (I_to_, I_Kr_, I_Ks_) to shorten APD (37). Clinically oriented experimental-computational studies and population-based modeling further support an inverse relationship between Ca^2+^ loading and APD across mammalian preparations, largely mediated by Ca^2+^-dependent regulation of I_Ca,L_, and secondary alterations in electrogenic Ca^2+^ transport, including Na^+^/Ca^2+^ exchange and Ca^2+^-ATPase activity, linking Ca^2+^ overload to both earlier repolarization and increased risk of Ca^2+^ mediated triggered activity (38–40).

Multiple *ATP1A3* pathogenic variants have been associated with human disease, including AHC (41). AHC presents in infancy with epilepsy, episodic hemiplegia, autonomic dysfunction, dystonia, nystagmus, and sudden unexpected death. Variants in *ATP1A3* account for 70% of reports of AHC and the D801N missense-causing variant is the most recurrent, associated with 30%–40% of instances of AHC (19). While this genetic association is known, the cause of sudden death in these patients remains unclear. Individuals with AHC have been viewed as at-risk for SUDEP, which is postulated to have multifactorial etiologies: neurologic, cardiovascular, and respiratory as well as iatrogenic (42). While the contribution of each factor to instances of SUDEP is not well understood, epilepsy can coexist with heritable arrhythmic syndromes (43). In a study of patients with SUDEP, 11% had a postmortem genetic finding of pathogenic/likely pathogenic variants in Na^+^ or K^+^ channel encoding genes, and many patients were found to have variants of uncertain significance in cardiac channelopathy-associated genes (42). Thus, genetic variants in genes that are responsible for the depolarization and repolarization of the cardiac myocyte may be common causes of SUDEP. Although there are no FDA-approved NCX1 modulating therapies, multiple ORM1-10103 analogs have been investigated in cardiac arrhythmia models (44), suggesting an important target of cardiac pathophysiology.

There is a shared substrate of risk in patients with ATP1A3-D801N, reflected in both a neurologic and cardiac risk of sudden death (45–47). For example, QTc variability has also been shown to transiently occur in patients with epilepsy during the ictal and post-ictal states (48). Given that ATP1A3-D801N causes seizures as well as dysautonomia in AHC, it raises the possibility of a central exacerbation of underlying myocardial arrhythmia susceptibility. A recent study in Atp1a3^D801N^ mice, which has exceedingly low expression of Atp1a3 in the heart, demonstrated a prodromal arrhythmic period that occurred before the pre-ictal window of terminal seizures, suggestive of an additional susceptibility to arrhythmia from dysautonomia alone (49).

Further studies should explore the autonomic regulation of cardiac arrhythmias in ATP1A3-D801N. In a recent preprint, we have shown that patients with ATP1A3-D801N exhibit paradoxical QT and QTc shortening at lower heart rates (50). We have also demonstrated that Atp1a3^D801N^ mice have increased sinus arrhythmia and sinus pauses in the early pre-ictal states (49). Consistent with these findings, other investigators have also shown that gain-of-function variants in other SQTS genes, such as the anion exchanger *SLC4A3*, also lead to paradoxical QTc shortening (51) and that augmented vagal effects may trigger ventricular arrhythmias in SQTS (52). We hypothesize that sinus pauses and bradycardia may precipitate ectopic activity and increase the arrhythmic vulnerability of a myocardium expressing ATP1A3-D801N. If validated, this mechanism may be relevant to other forms of SQTS.

### Limitations.

Our current study is limited by the immaturity of iPSC-CMs, which lack well-developed T-tubules. Subcellular localization, particularly within the T-tubule system, may be key to understanding ATP1A3 function. In murine hearts, Atp1a2 is known to preferentially express at T-tubules, while Atp1a1 shows no specific localization within the sarcolemma (53). To address this, primary adult cardiomyocytes isolated from human tissue, as well as engineered heart tissues and organoids, may be better suited for investigating the subcellular localization of ATP1A3 relation to ATP1A1-2 and NCX1.

*ATP1A3* constitutes roughly 26% of total NKA alpha subunit transcripts in cardiomyocytes, but a larger apparent loss of NKA function than observed here in the ATP1A3-D801N cells (e.g., on APD), according to the ToR-ORd simulations, suggests that the D801N missense variant may also impair the activity of the WT allele. In our previous international, multicenter case-control study, we observed that patients with heterozygous loss-of-function variants such as splice variants or *ATP1A3* deletions as well as other missense variants that are not D801N did not display QTc shortening (18), supporting the hypothesis that D801N may act through a different mechanism, possibly a dominant-negative mechanism. This interpretation is consistent with experimental evidence from *Xenopus laevis* oocytes expressing human *ATP1A3* transcripts (54). Although our mechanistic work did not directly assess the effect of the missense allele on the function of WT subunits, future studies using antisense oligonucleotides as a potential rescue approach could help address this important gap.

The autonomic nervous system findings in both patients with AHC and Atp1a3^D801N^ mice underscore a critical gap in our understanding of neuro-cardiac interactions. The potential role of autonomic abnormalities in patients with AHC leading to increased risk for arrhythmias in the setting of short QT remains unknown. We hypothesize that impaired autonomic regulation, either peri-ictal or in the setting of AHC-mediated bradycardia, may provide a “second hit,” which exacerbates the arrhythmic predisposition imparted by a shortened QT interval. Further studies are needed to explore this hypothesis. Importantly, animal models such as mouse, rat, sheep, *C*. *elegans, Drosophila,* and zebrafish, either express very low levels of Atp1a3 in the heart or lack a cardiac ortholog entirely. This lack of ATP1A3 ortholog in commonly used animal models has limited our ability to address the translations of our findings in an animal model because the animals do not adequately recapitulate the cardiac pathology that manifests in humans harboring ATP1A3-D801N.

Additionally, systemic inhibition of NCX1 may affect exchanger function in noncardiac tissues, making it important to understand potential off-target effects of this therapeutic strategy. Because we lacked a mouse model with endogenous cardiac Atp1a3 expression, we were unable to evaluate the effect of ORM-10103 on other organ systems. Nonetheless, our findings provide proof of concept. Further studies should focus on models that more faithfully recapitulate human ATP1A3 expression in the myocardium, enabling assessment of NCX inhibition beyond the heart. These may include coculture systems using patient-derived iPSC cardiomyocytes and neurons to address autonomic abnormalities found in this disease, as well as transgenic mice engineered to express cardiac Atp1a3 at human-comparable levels.

In conclusion, our findings contribute to the growing evidence linking ATP1A3 to channelopathies, the phenotypic expansion of AHC, and the possible mechanistic link between SQTS with SUDEP.

## Methods

### Sex as a biological variable.

This study uses cell lines from 1 female AHC patient and 1 male AHC patient, and similar findings were observed in these lines.

### Statistics.

For all experiments, a minimum of 3 passages were used for each line per experiment. To determine statistical significance among groups, a paired 2-tailed Student’s *t* test was performed for data with a normal distribution and 2 groups, and a 1-way ANOVA with multiple comparisons was used for 3 groups. For statistical tests assuming a normal distribution, the Shapiro-Wilk test for normality was used. For nonparametric data, a Mann-Whitney *U* test was used when comparing 2 groups. Fisher’s exact tests were used for categorical data. Mann-Whitney *U* tests or Wilcoxon matched-pairs tests were conducted for nonparametric statistical analysis for whole cell patch clamp experiments. Live cell imaging experiments were analyzed using a hierarchical statistical approach, as previously described (55). With nonparametric data, the data were first log_10_-transformed before applying the R code. Data are presented as the mean ± SEM. *P* < 0.05 were considered statistically significant.

### Study approval.

This study was approved by Duke University IRB (Pro00056651 and Pro00094341). Informed consent was obtained from each participant or participant’s parent/legal guardian in accordance with the Declaration of Helsinki. Methods are further detailed in the Supplemental Methods.

### Data availability.

A detailed description of all experimental methods can be found in the Supplemental Methods. This study reanalyzes previously published RNA-seq data as detailed in the Supplemental Methods. Supporting data values of relevant panels are provided in the Supporting Data Values file. Raw data are available upon reasonable request to the corresponding author.

## Author contributions

Design and interpretation of results were contributed by MTKB, PM, ZZ, DR, DN, BS, RMP, MEMM, PKRM, ASH, AH, CQS, ALG, DMB, NB, MAM, and APL. Supervisory roles were contributed by ALG, DMB, NB, MAM, and APL. Data acquisition was contributed by MTKB, PM ZZ, DR, DN, BS, MEMM, and PKRM. Writing of manuscript was contributed by MTKB, PM, RMP, ALG, DMB, and APL.

## Funding support

This work is the result of NIH funding, in whole or in part, and is subject to the NIH Public Access Policy. Through acceptance of this federal funding, the NIH has been given a right to make the work publicly available in PubMed Central.

- The Paul Gillette PACES Research Grant (MTKB.)
- National Institute of Health (NIH) T32 GM007171 (MTKB).
- NIH (R01-HL160654, R01-HL166217) (APL).
- Doris Duke Charitable Foundation (CSDA-2020098) (APL).
- John Taylor Babbitt Foundation (APL).
- The Hartwell Foundation (APL).
- Additional Ventures (APL).
- Y.T. and Alice Chen Pediatric Genetics and Genomics Research Center (APL).
- NIH (NS125785) (ALG).
- Alternating Hemiplegia of Childhood Foundation (ALG).

## Supplementary Material

Supplemental data

Unedited blot and gel images

Supporting data values

## Version 1 04/08/2026

Electronic publication
