# Supplementary material for: D801N in ATP1A3-encoded Na/K-ATPase alpha 3 causes cardiac arrhythmogenesis through sodium-calcium exchanger–mediated calcium overload
Source: JCI Insight. 2026 Apr 8;11(7):e197721. doi: 10.1172/jci.insight.197721 (PMC13134723; doi:10.1172/jci.insight.197721)
Supplement: Supplemental data [file jciinsight-11-197721-s086.pdf]

D801N in *ATP1A3*-encoded Na/K-ATPase Alpha 3 causes Cardiac Arrhythmogenesis through Sodium-Calcium Exchanger-mediated Calcium Overload

Minu-Tshyeto K. Bidzimou, B.A, Padmapriya Muralidharan, PhD, Zhushan Zhang, MD PhD, Danyal Raza, M.S, Daniel Needs, PhD, Bo Sun, PhD, Robin M. Perelli, PhD, Mary E. Moya-Mendez, MD, MS, P.K. Rakesh Manivannan, MBBS, Arsen S. Hunanyan, PhD, Abbigail Helfer PhD, Christine Q. Simmons, MD, Alfred L. George, Jr., MD, Donald M. Bers, PhD, Nenad Bursac, PhD, Mohamad A. Mikati, MD, Andrew P. Landstrom, MD PhD

SUPPLEMENTAL MATERIALS

SUPPLEMENTAL METHODS

ToR-ORd in silico model

To understand the impact of the Na^+/K^+ ATPase pump (NKA) on the action potential duration (APD) of cardiomyocytes, we employed the Tomek–Rodriguez–O'Hara–Rudy human cardiomyocyte *in silico* model (ToR-ORd)(1). A scaling factor was applied to the NKA current to simulate different NKA functional states. The levels chosen were 1X (control), 0.75X, 0.5X, 0.25X, and 0.1X. To pace the model, a stimulus current was applied at 1Hz for 1000 total paces to allow for dynamic adjustment of the intracellular ion concentrations. APD was measured as established in the model. Steady state inactivation of the L-type Ca^{2+} channel was calculated as combination of voltage-dependent and calcium-dependent mechanisms using a steady state sigmoidal function. We used the inactivation variable:

$F(V,[Ca]_i)= 1/(1+\exp((V-V_{1/2})/k)))$

Where k=3.696 mV, and the half inactivation voltage $V_{1/2}$ was calculated as

$V_{1/2}=V^0_{1/2}- \Delta V^* \log([Ca^{2+}]_i/[Ca^{2+}]_{ref})$

With $V^0_{1/2}$= -19.59, ΔV = 15.

$V^0_{1/2}$ and k were obtained from the fss equation in the ToR-ORd model. ΔV was set empirically to 15mV per log unit increase in $[Ca^{2+}]_i$. Simulations were performed over a voltage range of -80 to +60mV, with $[Ca^{2+}]_i$ of each NKA functional states that are measured after 1000 paces.

Mapping of amino acid positions on crystal structure

Previously published short QTc-associated variants in ATP1A3(2) were mapped on the human ATP1A3 crystal structure in the potassium occluded state (PDB ID: 8D3X)(3) using PyMOL software (Shrodinger, Portland, USA).

RNAseq Analysis

Meta-analysis of previously published RNAseq data sets on human myocardium were acquired to determine relative transcript abundance of *ATP1A* isoforms. Datasets were downloaded from the Gene Expression Omnibus (GEO) database. The following human cardiomyocyte datasets were used: GSE116250, GSE123976, GSE46224, GSE57344, GSE55296, GSE126573, SE71613, PMID 31155234, and GSE183852. A previously published murine dataset from our lab was also analyzed(4). The following rat datasets were used: GSE197999, PMID 29231921. The following sheep dataset was used: PMID 22508961. Percentages were calculated by totaling mean counts of *ATP1A1-3* and dividing each *ATP1Ax* by the total mean count for each sample.

Human induced pluripotent stem cells

This study received approval from the Institutional Review Boards at Baylor College of Medicine and Duke University Health System and uses previously established induced pluripotent stem cell lines (iPSCs)(5). Two lines were derived from patients with the ATP1A3-D801N missense (iPSC^{D801N} and iPSC$^{D801N-2}$). The iPSC^{D801N} missense was

corrected using CRISPR to generate an isogenic control (iPSC^{cWT}). An additional WT line from a healthy individual was used (iPSC^{WT-2}).

Differentiation of iPSC-CMs

IPSCs seeded on Matrigel coated wells were selected for differentiation to generate iPSC-derived cardiac myocytes (iPSC-CMs) when 90% colony confluence was achieved. iPSCs were differentiated into iPSC-CMs utilizing an adapted method(6). On day 0 of differentiation, 60ng/ul Activin A (StemCell Technologies, Vancouver, Canada), 12uM CHIR-99021 (Cayman Chemical, Ann Arbor, USA), 50ug/ml Ascorbic Acid (Tocris Bioscience, Bristol, United Kingdom) in RPMI 1640 with 2% B27 without insulin (Life Technologies, Carlsbad, USA) (RPMI/B27-) was added. On days 1 and 2, the media was replaced daily with 5uM IWR1 (StemCell Technologies, Vancouver, Canada) and 50ug/ml Ascorbic Acid in RPMI/B27-. On day 4, the media was changed to RPMI/B27- with 5uM IWR1. On day 6 the cells were fed maintenance media, which composed of RPMI 1640 with 2% B27 with insulin (Life Technologies, Carlsbad, USA) and monitored for the onset of beating. Cells were continually fed with maintenance media every 2 days until day 15, and 3 times per week thereafter. Cardiomyocytes were used for experimentation between D40-D60 since differentiation.

ATP1A3 expression constructs

Vectors containing full-length, wild-type, human *ATP1A3* sequence corresponding to the reference sequence were obtained (pRP[Exp]-EGFP-CAG>hATP1A3, [NM_152296.4],

cat# VB180814-1968xpj, Vector Builder). Vectors containing the D801N mutation in *ATP1A3* were created using site-directed mutagenesis (Cat# 200518, Stratagene, San Diego, USA). A ouabain-resistant ATP1A3 expression construct was created using site directed mutagenesis (Cat# 200518, Stratagene, San Diego, USA) to insert 2 point mutations (Q108R, N119D) that haven been shown to maintain pump function in the presence of ouabain (ATP1A3OR)(7). Sanger sequencing was used to validate mutation insertions as previously described.

HEK293T transfection

To determine the impact of the D801N variant on the function of ATP1A3, WT, D801N, and ouabain-resistant expression constructs were expressed following transfection in human embryonic kidney 293T (HEK) cells using the Lipofectamine^{TM} 3000 Reagent Protocol (Invitrogen Thermofisher Scientific, Waltham, USA). Cells were incubated in the presence or absence of 10uM ouabain for 48 hours with daily media changes. Cell viability was quantified after 48hrs.

Immunofluorescence

IPSC-CMs were split onto Matrigel coated 35mm dishes with a 14mm microwell (Cellvis, Cat# D35-14-1.5-N, Mountain View, USA) using the StemDiff cardiomyocyte dissociation kit (Cat# 05025, STEMCELL Technologies, Cambridge, USA). Cells were fixed using 4% PFA, permeabilized with 0.5% Triton X-100, incubated with primary antibodies (Invitrogen, Carlsbad, CA), and detected using Alexa Fluor-conjugated secondary antibodies

(Jackson ImmunoResearch, West Grove, USA). Primary antibodies (1:400 dilution) used include mouse anti- human cardiac troponin T (Cat# MA1-20112, Invitrogen, Waltham, USA), rabbit anti-human ATP1A3 (Cat# 06-172-I, Millipore Sigma, Burlington, USA), and mouse anti-human NCX1 MA3-926, ThermoFisher, Waltham, USA). Image acquisition was performed on an Olympus IX50 fluorescence microscope (Olympus Corporation, Tokyo, Japan) and analyzed with Fiji ImageJ v.2.14.0 (National Institutes of Health, Bethesda, USA).

Wheat germ agglutinin staining

IPSC-CMs were fixed using 4% PFA, and 5ug/mL wheat germ agglutinin in PBS was applied to cells for 10 min in the dark before permeabilization. Following permeabilization, cells were stained with primary antibodies for immunofluorescence as described above.

Western blot

IPSC-CMs were homogenized with RIPA (ThermoFisher Scientific, Waltham, USA) in the presence of protease and phosphatase inhibitors (ThermoFisher Scientific, Waltham, USA). The supernatant was collected after centrifugation at 16,000 x g for 10 min at 4°C. Protein concentration was measured by NanoDrop (ThermoFisher Scientific, Waltham, USA) and 30µg of protein was run on 4-15% acrylamide gels (Bio-rad, Hercules, USA). Gels were transferred to methanol-activated PVDF membranes (Bio-rad, Hercules, USA) and blocked in 5% non-fat milk/TBST. Primary antibodies included rabbit anti-human ATP1A3 (cat# 06-172-I, Millipore Sigma, Burlington, USA) and mouse anti-human GAPDH (Cat# MA5-15738, ThermoFisher Waltham, USA), mouse anti-human NCX1

(cat# MA3-926, ThermoFisher, Waltham, USA), rabbit anti- human ATP1A2 (cat# ab166888, Abcam, Waltham, USA), mouse anti-human ATP1A1 (cat# ab7671, Abcam, Waltham, USA), and mouse anti-human ATP1B1 (cat# MA3930, ThermoFisher, Waltham, USA). Antibodies were visualized using anti-mouse or anti-rabbit HRP secondary (1:1000) and chemiluminescence was activated with ECL substrates (Bio-Rad, Hercules, USA). Images were acquired using the ChemiDoc MP Imaging System (Cat# 12003154,Bio-Rad, Hercules, USA).

Arclight Transduction

IPSC-CMs were split onto Matrigel coated 35mm dishes with a 14mm microwell (Cellvis, Cat# D35-14-1.5-N, Mountain View, USA) using the StemDiff cardiomyocyte dissociation kit (cat# 05025, STEMCELL Technologies, Cambridge, USA). During this process, 10^8 TU/mL Arclight Lentivirus (pLV[Exp]-EF1A>(8); Cat #VB220422-1240vdf, Vectorbuilder, Chicago, USA) was diluted 1:1000 in CM support media. The CM pellets were then resuspended with the lentivirus loaded CM support media at a final concentration of 10^6 cells/mL and plated on the coated 35mm dishes. After 24hrs, media was changed to RPMI/B27+ and experiments were conducted 48hrs post transduction.

Live-cell imaging

Live-cell imaging was conducted to determine intracellular Ca^{2+} dynamics and membrane polarity in iPSC-CMs. Cells were split onto Matrigel coated 35mm dishes with a 14mm microwell (Cat# D35-14-1.5-N, Cellvis, Mountain View, CA) using the StemDiff

cardiomyocyte dissociation kit (Cat# 05025, STEMCELL Technologies, Cambridge, USA). For Ca^{2+} transient and Ca^{2+} leak measurements, iPSC-CMs were incubated with 10 μM CAL-520 (ab171868, Abcam, Waltham, USA) in Ca^{2+}-free Tyrode for 1 hour. After washing with Ca^{2+}-free Tyrode, cells were incubated in a 1:1 solution of Tyrode (with 1.8mM $CaCl_2$) and RPMI/B27$^+$ for 15 mins before experiments. To measure intracellular Ca^{2+} transients, leak in Cal-520 loaded cells, and fluorescence traces in Arclight-transduced cells, linescan images were obtained on a Zeiss Laser Scanning Confocal 510 Meta Microscope (Carl Zeiss AG, White Plains, USA) at 0.1μm per pixel. Lines were drawn on the longitudinal axis of iPSC-CMs. Arclight transduced cells were pre-paced at 0.5Hz for using an IonOptix MyoPacer field stimulator (Ionoptix LLC, Westwood, USA) for 5 min (17.0V, 10msecs/stimulus), and then recorded at their spontaneous depolarizing rate for analysis for 30 secs each. CAL-520 loaded cells were paced at 0.5Hz with an IonOptix MyoPacer field stimulator for 10 secs (17.0V, 10msecs/stimulus) followed by 20 seconds of diastole recording. Fluorescence traces (representing action potential) and Ca^{2+} transients were analyzed with Fiji ImageJ v.2.14.0 (National Institutes of Health, Bethesda, USA). Ventricular iPSC-CMs were identified by action potential morphology with clear phase 2, measured as ratio >0.6 of APD50/APD90). Sparkmaster 2 was used to identify Ca^{2+} sparks and miniwaves(9). Sparks were measured 2 seconds after the last Ca^{2+} transient, for 4 secs. Spark width, amplitude (F_{max}/F_0), duration, and frequency of sparks was quantified. Ca^{2+} leak was calculated using the following equation: leak = width* amp* dur* freq. Leak for each line was further normalized to their respective mean Ca^{2+} SR store. To measure SR Ca^{2+} store, 10mM Caffeine was added to the culture under real-time imaging to trigger the release of stored Ca^{2+}. Peak fluorescence following

caffeine addition was measured as an indication of stored Ca^{2+}. Intracellular [Ca^{2+}] was measured using Fura-2AM (Cat# F1221, ThermoFisher, Waltham, USA). In each well, 5uM Fura-2 in 0.1% Pluronic Tyrode solution without Ca^{2+} was added and incubated for 20 mins. Each well was washed with Tyrode and a 2mL 1:1 mixture of RPMI/B27+:Tyrode was added, and cells were incubated for an additional 15 mins before imaging. Cells were paced at 0.5Hz using an IonOptix MyoPacer field stimulator (Ionoptix LLC, Westwood, USA) for 10 secs (17.0V, 10msecs/stimulus). Cells were excited at 340nm and 380nm. Fluorescence was recorded at 510nm with a NiKON Eclipse TE2000-E and measured using the fluorescence ratio imaging software Metafluor. For rescue experiments, cells were incubated in 10^{-5}M ORM-10103 in RPMI/B27+ media prior to live cell imaging. Activity of SERCA2a was calculated as the difference between the rate of decay (K) of the spontaneous Ca^{2+} transient, which reflects Ca^{2+} removal from the cytosol by the combined activities of SERCA2A and NCX, and the K of the caffeine-induced transient which reflects Ca^{2+} extrusion by NCX only(10, 11). The following equations were used:

If K = 1/tau, $K_{caffeine}$ = NCX activity, and $K_{spontaneous}$ = SERCA2A activity + NCX activity, then NCX activity = $1/tau_{caffeine}$ and SERCA2A activity = $1/tau_{spontaneous} - 1/tau_{caffeine}$.

Action potential recordings

IPSC-CMs were split onto Matrigel coated cover slips using the StemDiff cardiomyocyte dissociation kit (Catalog# 05025, STEMCELL Technologies, Cambridge, USA). Action potentials were recorded in whole cell current-clamp mode using a MultiClamp-700A amplifier with Digidata 1322A interface and Clampex 8 software (Axon Instruments, Union City, USA). The cover-glass with mounted CMs was transferred to a chamber mounted

on an inverted microscope (OLYMPUS IX50) and continuously perfused (at 35-37°C, 2ml/min) with Tyrode's solution containing (in mM): 140 NaCl, 5.4 KCl, 2 CaCl2, 1 MgCl2, 10 HEPES, 10 glucose, pH 7.4. The patch pipettes had a resistance of 6~10 MΩ when filled with pipette solution containing (in mM): 130 K-aspartate, 6 NaCl, 3 Mg-ATP, 0.4GTP,10 HEPES, 5 EGTA, and pH 7.2 (with KOH). Action potentials were recorded in spontaneously beating iPSC-CMs, or elicited by depolarizing current injection (6ms, 1.5 x threshold, 0.5Hz) in non-beating cells. Clear inward sodium and calcium channel current was confirmed with a pre-test voltage-clamp ramp protocol (-100 to +100mV, 1mV/ms). Data were acquired at sample rate of 10 kHz (filtered at 1 kHz) and analyzed with pCLAMP 10 software (Axon Instruments). AP morphology and AP parameters were analyzed as previously described(12, 13). Cardiomyocyte with ventricular-like AP were selected based on presence of a distinct plateau phase (phase 2, measured as ratio >1.5 of APD30-40/APD70–80). Pharmacologic treatments included application of 10^{-7}M ouabain (cat# B3436, ApexBio, Houston, USA) and 10^{-5}M ORM-10103 (cat# SML0972, Sigma Millipore, Rockville, USA).

Sodium-calcium exchanger current

I_{NCX} was recorded using a voltage-clamp protocol as previously described(14). iPSC-CM cells are perfused by external solution at 35-37°C, in which Na^+ channel, Ca^{2+} channel, K^+ channel and Na^+/K^+ pump currents are blocked. External solution component (in mM): 140 NaCl; 1.0 MgCl2; 5.0 HEPES; 2.5 CaCl2; 1.0 BaCl2; 10 glucose; 0.01 Nitrendipine, & 0.01 Strophanthidin; pH7.4, w/ NaOH. Pipette solution containing (in mM): 110 CsCl; 20 TEACl; 10 NaCl; 0.4 MgCl2; 10 HEPES; 1.0 CaCl2; 5.0 glucose; 5.0 EGTA; pH7.2, w/

CsOH; 1.5~3.5MΩ. I_{NCX} are induced by ramp protocol of 2 sec from +80mV to -120mV, HP-80mV, every 10sec. NiCl2 (5mM) was added. I_{NCX} is measured as the Ni-sensitive current. To quantify ion flux from current density, a standard electrophysiological approach was employed to relate transmembrane current to ionic movement as previously described(15).

L-type Ca^{2+} channel current

L-type Ca^{2+} channel (LTCC) current was recorded with the whole-cell patch-clamp technique as previously described with some modifications(12, 16, 17). Voltage-clamp experiments were performed with an Axopatch 700A amplifier interfaced with Digidata 1550b(Molecular Devices, San Jose, USA). pClamp 10 &11 softwares were used for data acquisition and analysis. Data were filtered at 1 kHz and sampled at 10 kHz. The pipette resistances were 6-10 MΩ when filled with pipette solution containing (in mM):92 K-aspartate, 48 KCl, 10 HEPES, 0.02 EGTA, 0.1 GTP-Tris, 1 MgATP, 4 Na_2ATP . (pH 7.2 with KOH). IPSC-CMs on cover-glass were perfused with external solution containing (in mM): 140 NaCl, 10 Glucose, 10 HEPES, $CaCl_2$ 2, 1 $MgCl_2$, 4 KCl, 5 Aminopyridine and 0.1 $BaCl_2$(pH 7.35 with NaOH) at 35-37°C. After formation of giga-ohm seal and break through cell membrane, the $I_{Ca,L}$ peak currents were recorded by holding the iCMs at -80mV followed by a pre-conditioning pulse at -40mv to inhibit Na^+ channel current and possible T-type Ca^{2+} current followed by a 100msec test pulse at 0mv. LTCC was elicited by a testing potential of 10mV for 300ms once every 10 second with a 50ms pre-pulse of -40mV to 0mV. LTCC was measured and normalized by membrane capacitance (pA/pF) for cell size. Current densities were compared between the WT and mutant iPSC-CMs.

The time duration (tau) for the decay phase of $I_{Ca,L}$ peak currents were measured using Levenberg-Marquardt fitting method.

Statistical analysis

For all experiments, a minimum of 3 passages were used for each line per experiment. To determine statistical significance among groups, a paired Student's t-test was performed for data with a normal distribution and two groups, and a one-way ANOVA with multiple comparisons was used for 3 groups. For statistical tests assuming a normal distribution, the Shapiro-Wilk test for normality was used. For non-parametric data, a Mann-Whitney test was used when comparing two groups. Fisher's exact tests were used for categorical data. Mann-Whitney tests or Wilcoxon matched-pairs tests were conducted for non-parametric statistical analysis for whole cell patch clamp experiments. Live cell imaging experiments were analyzed using a hierarchical statistical approach, as previously described(18). With non-parametric data, the data was first Log10-transformed before applying the R code. Data are presented as the mean ± SEM. $P<0.05$ were considered statistically significant.

SUPPLEMENTAL RESULTS

HEK-293T ATP1A3 constructs overexpression

We transfected HEK293T cells with full-length human *ATP1A3* with and without the D801N missense and compared cell survival. *ATP1A3* overexpressing cells had

comparable expression to empty vector and ATP1A3-D801N encoding transcripts. To determine whether the presence of the D801N mutation affects the pharmacologic impact of ouabain inhibition, we created a ouabain resistance dual mutation construct which is not able to bind ouabain for both WT and D801N ATP1A3(19) (ATP1A3^{OR} and D801N^{OR}, respectively) and quantified survival. As expected, addition of ouabain to the media caused significant cell death in empty vector transfected HEK293T cells, while ATP1A3^{OR} cells demonstrated increased survival. Conversely, cells transfected with D801N^{OR} had significant reduced survival compared to ATP1A3^{OR} which was equivalent to EV. Together, these findings suggested that the D801N variant created a loss-of-function effect on ATP1A3 in HEK293T cells. These findings are consistent with previous report which have linked ATP1A3 overexpression with apoptosis and autosis (20, 21).

ATP1A1-3 **expression levels across species**

We used a comparative genomic analysis of human and *Caenorhabditis elegans* known as OrthoList 2 to determine the orthologs as well as the cardiac specific transcription of *ATP1A1-3(22).* We found that *eat-6* encodes for the catalytic peptide of a P-type Na^+/K^+ exchanging transporter, and is an ortholog of *ATP1A1, ATP1A2,* and *ATP1A3,* such that it is not a specific one-to-one orthologue. It is transcribed in several structures, including the pharynx, which is analogous to vertebrate cardiac tissue. Similarly, in *Drosophila melanogaster, Atp*α encodes for a P-type Na^+/K^+ exchanging transporter but does not have a one-to-one ortholog to human ATPA1-3 isoforms. It translates a protein with 76.3% homology to ATP1A3 and is expressed in cardiomyocytes(23). In Zebrafish, *atp1a3a* and *atp1a3*b are the *ATP1A3* orthologs but they are not transcribed in the cardiovascular

system, according to the Zebrafish Information Network (ZFIN) database. In the heart, only *atp1a1* is transcribed and is an ortholog of *ATP1A1*.

SUPPLEMENTAL FIGURES

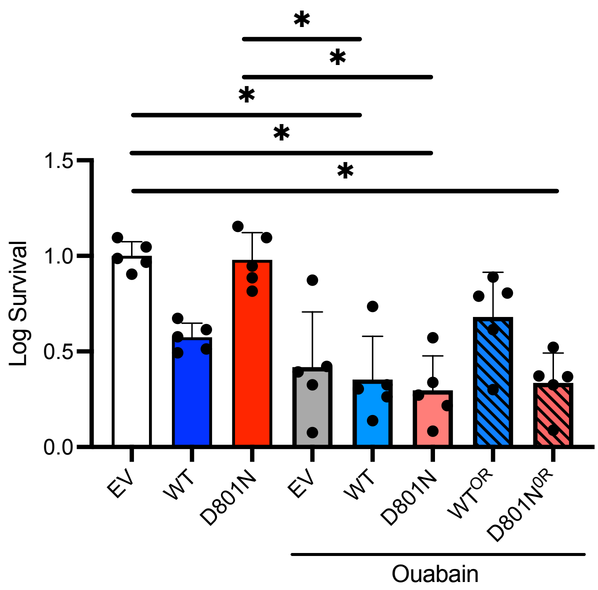

Supplemental Figure 1: Transfection of HEK-293T cells and survival analysis. Cells were transfected with EV (empty vector), ATP1A3 (WT), or ATP1A3-D801N (D801N) constructs, and cell survival was measured and normalized to EV. In the presence of ouabain, cells were transfected with EV, ATP1A3, ATP1A3-D801N, ouabain resistant (Q108R, N119D) ATP1A3 (WT^{OR}), or ouabain resistant ATP1A3-D801N ($D801N^{OR}$). A Kruskal-Wallis test was employed, * indicates $p<0.05$.

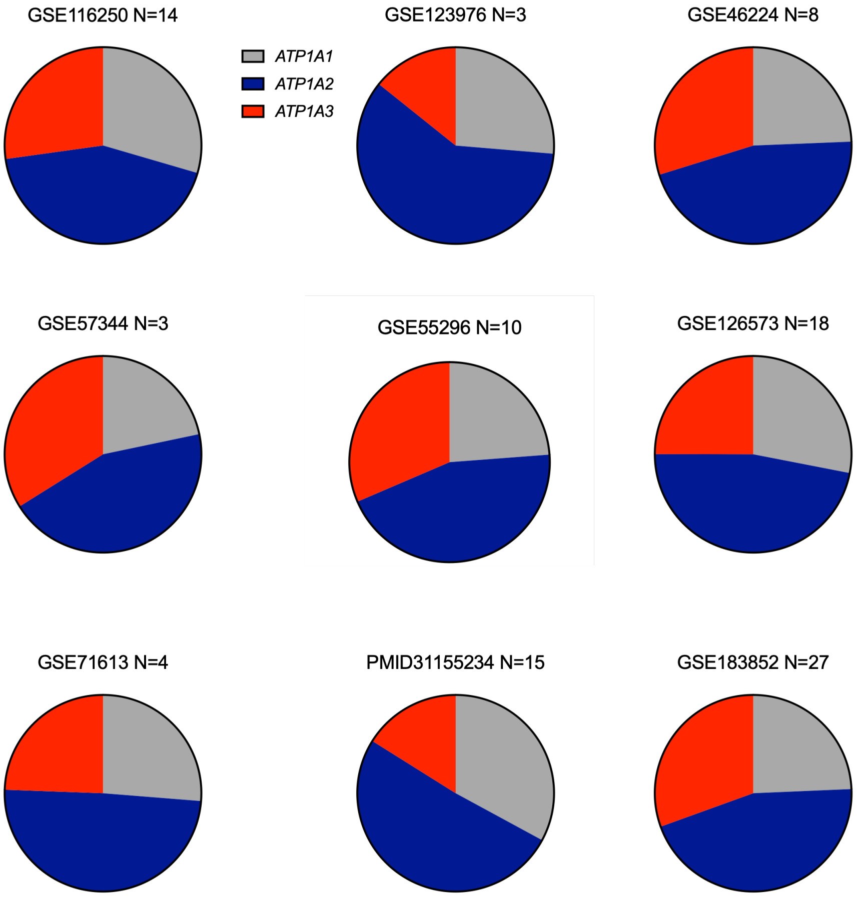

Supplemental Figure 2: *ATP1A1-3* relative transcription levels in human myocardium. Pie charts illustrating relative transcription levels of *ATP1A1-3* isoforms in 9 studies using human myocardium. N indicates number of samples per study.

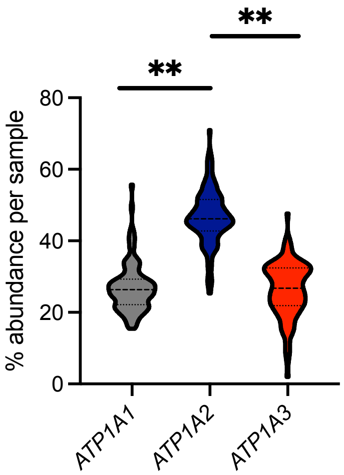

Supplemental Figure 3: Relative abundance of human *ATP1A1-3* isoforms per sample in published databases. Violin plot illustrating *ATP1A1-3* transcription in each human cardiomyocyte sample analyzed from published RNA sequencing data in relative abundance by percentages. Total number of samples in this analysis, N=102. Average relative transcription levels of *ATP1A1-3 are* 26.97%, 46.65%, and 26.34%, respectively. Repeated measure one-way ANOVA with the Geisser-Greenhouse correction and Tukey's multiple comparison tests were conducted. ** indicates $p<0.01$.

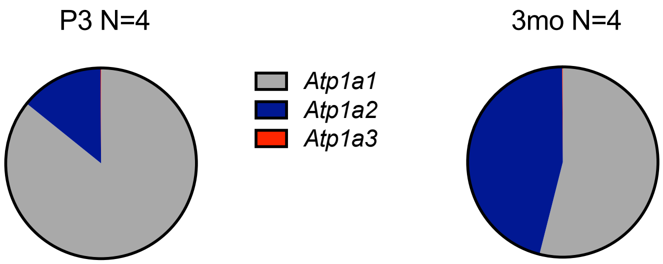

Supplemental Figure 4: Relative transcription of *Atp1a1-3* in C57/Black6J WT mice. Pie charts illustrating relative abundance of transcripts in samples collected at P3 and 3 months. N=4 samples.

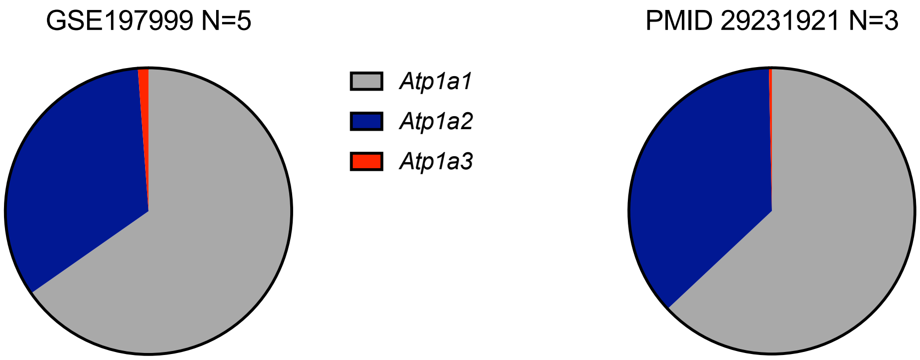

Supplemental Figure 5: *Atp1a1-3* relative transcription levels in rats. Pie charts illustrating relative transcription levels of *Atp1a1-3* isoforms in 2 studies using rats. N indicates number of samples per study.

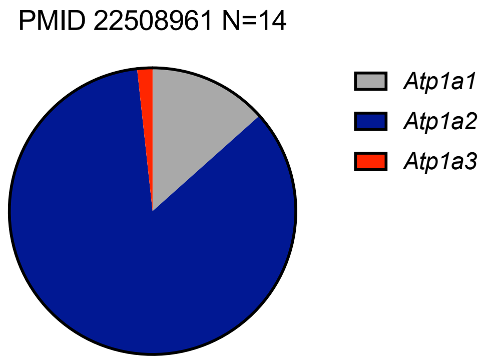

Supplemental Figure 6: relative transcription of *Atp1a1-3* in sheep. Pie chart illustrating relative abundance of transcripts in samples collected in sheep hearts. N indicates number of samples in study.

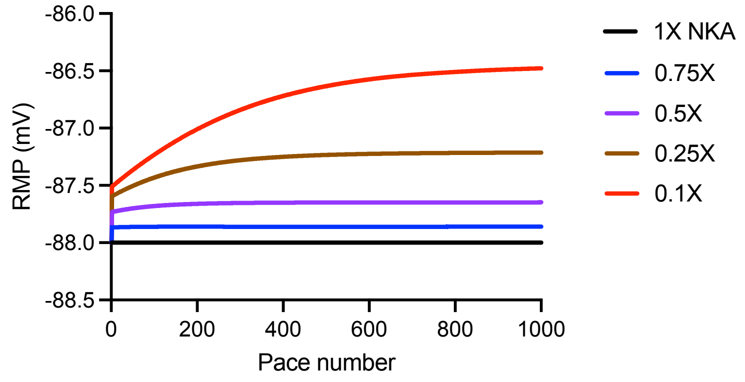

Supplemental Figure 7: Simulated resting membrane potential. Graph illustrating resting membrane potential (RMP) as a function of myocyte pace number in a set of simulations with different Na^+/K^+ ATPase (NKA) functional states in the ToR-ORd model.

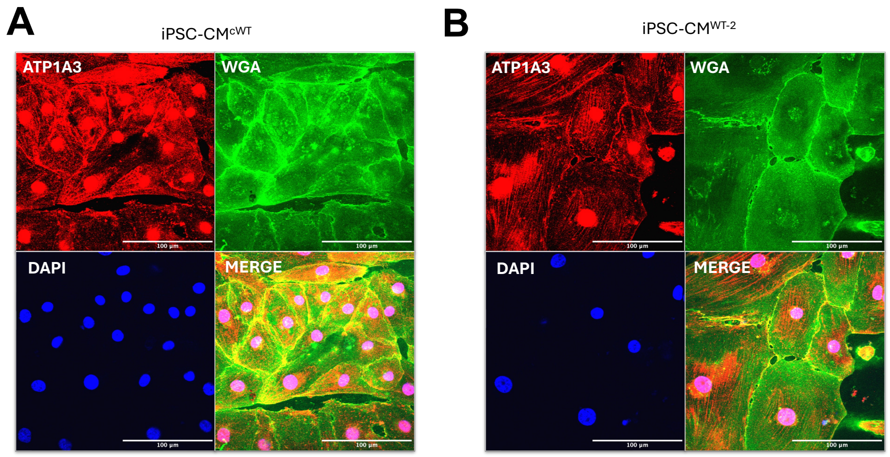

Supplemental Figure 8: membrane localization of ATP1A3 in iPSC-CMs^{WT}. Representative immunofluorescence images of two independent WT iPSC-CM lines, iPSC-CM^{cWT} (Panel A) and iPSC-CM$^{WT\text{-}2}$ (Panel B). For each panel, ATP1A3 immunostaining is shown in red (top left), wheat germ agglutinin (WGA) staining to delineate the cell membrane is shown in green (top right), nuclear staining with DAPI is shown in blue (bottom left), and merged images are shown in the bottom right. ATP1A3 signal demonstrates prominent membrane localization and colocalization with WGA in both WT lines. Scale bar=100 microns

Commented [AL1]: Can you label the two panels A and B and define a bit more in the legend?

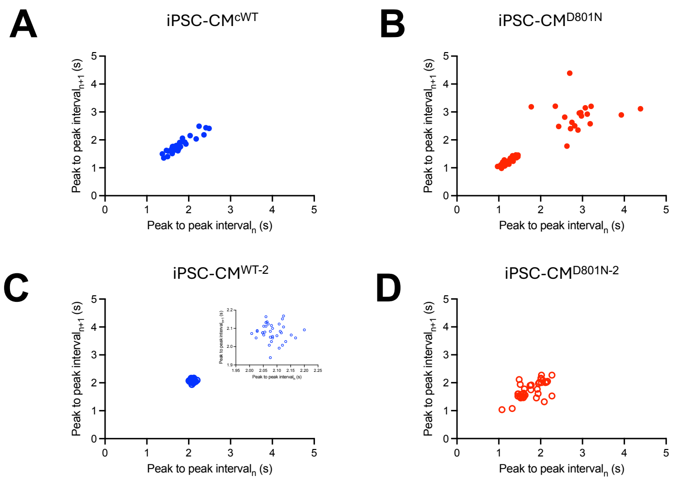

Supplemental Figure 9: Interval rate of spontaneously fluorescing iPSC-CMs. Bar graphs illustrate peak to peak interval fluctuation in WT and D801N myocytes. Panels A-D correspond to iPSC-CM^{cWT}, iPSC-CM^{D801N}, iPSC-CM^{cWT-2}, iPSC-CM$^{D801N-2}$, respectively, Points that deviate from a x=y relationship indicate irregular fluorescence amplitudes associated with delayed after depolarizations. Inset in panel C depicts a zoomed-in view of Panel C to facilitate visualization of individual data points.

Commented [AL2]: Here too, can you please label the panels A-D. Be sure to mention the inset in panel C in the legend?

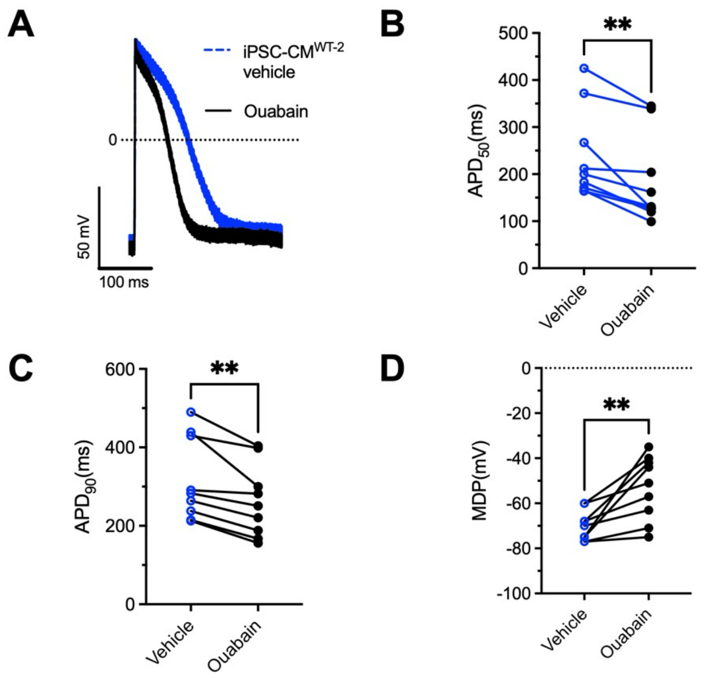

Supplemental Figure 10: Ouabain effect on action potential parameters in iPSC-CM^{WT-2}. Patch clamp recording of iPSC-CM^{WT-2} with application of vehicle or 10^{-7}M ouabain. A) Representative action potential traces. B) Action potential duration at 50% and C) 90% repolarization. D) Maximum diastolic potential. A Wilcoxon matched-pairs test was conducted. ** $p<0.01$.

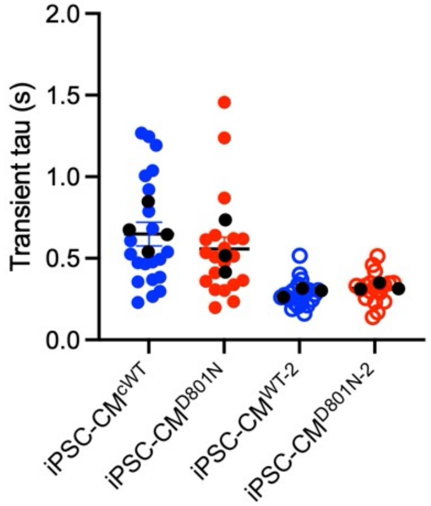

Supplemental Figure 11: Tau of Ca^{2+} transient amplitude. Graph shows fluorescence decay constant of Ca^{2+} transients for each cell. n= 21, 20, 23, and 19, respectively. Black dots represent experimental means. A hierarchical statistical test was conducted, and no difference was found between D801N lines and respective controls

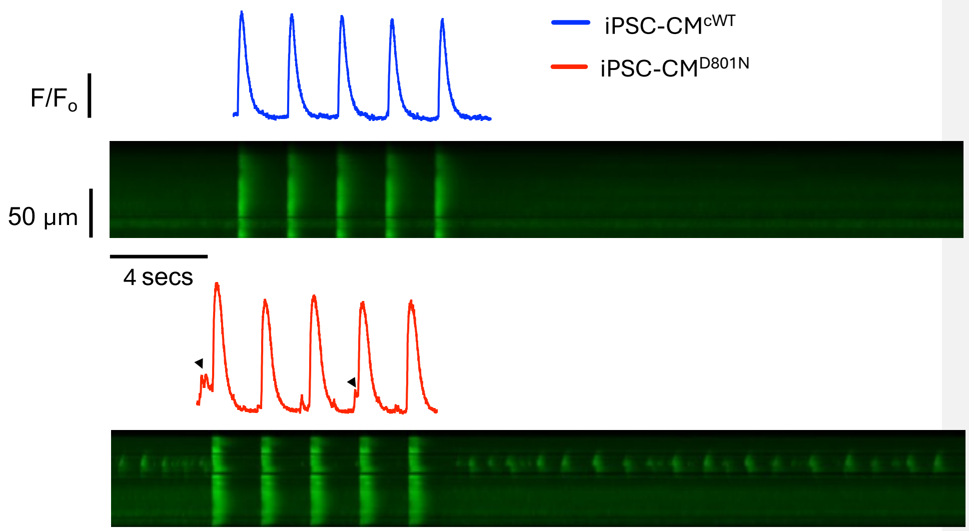

Supplemental Figure 12: Linescan images of Ca^{2+} transients. Representative linescan images of Cal-520 loaded iPSC-CM^{D801N} and iPSC-CM^{WT} cells paced at 0.5 Hz. Black arrow indicates miniwaves.

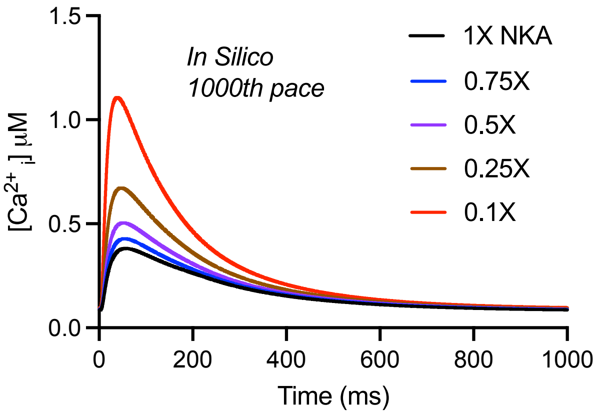

Supplemental Figure 13: Simulated intracellular Ca^{2+} in the ToR-ORd model of human ventricular cardiomyocyte. Graph illustrates $[Ca^{2+}]_i$ vs time on the 1000th pace in a set of simulations that use different NKA functional states.

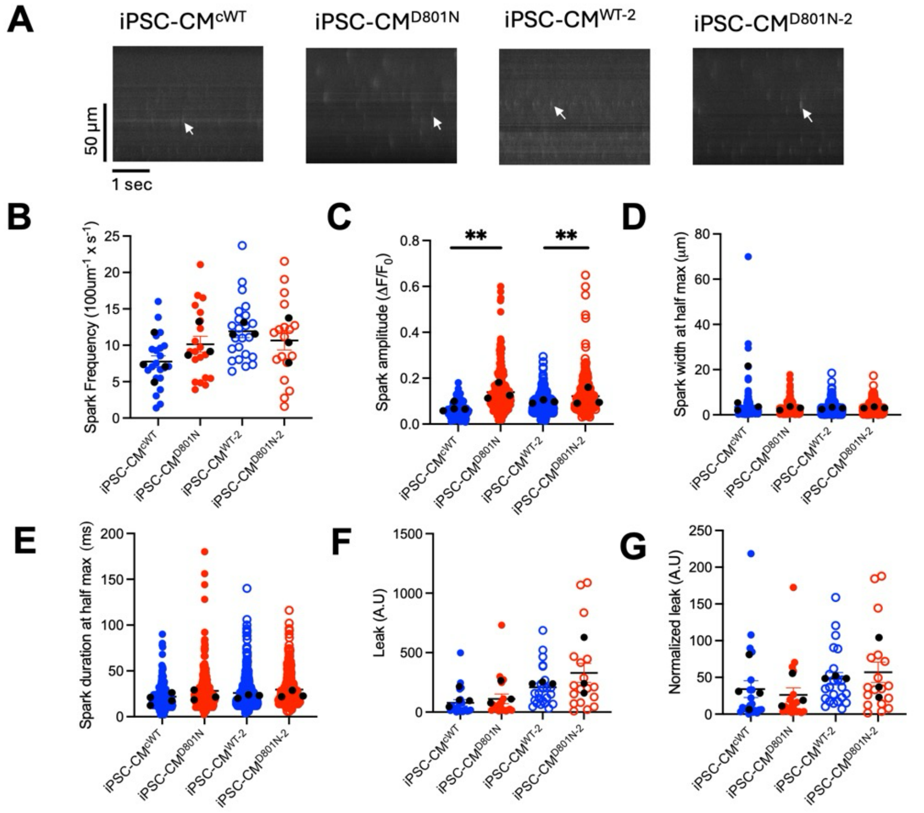

Supplemental Figure 14: Ca^{2+} sparks. A) Representative linescan images of Ca^{2+} sparks for iPSC-CMs^{WT} and iPSC-CMs^{D801N}. B) Frequency of sparks per cell among spark positive cells. (A-C) Spark amplitude, full width at half max, and full duration at half max, respectively. C) Calculated Ca^{2+} leak per cell (A.U= arbitrary unit). D) Calculated Ca^{2+} leak normalized to mean Ca^{2+} SR store amplitude. Sparks n=212, 313, 517, 343, respectively. Black dots represent experimental means. A hierarchical statistical test was conducted. ** $p<0.01$.

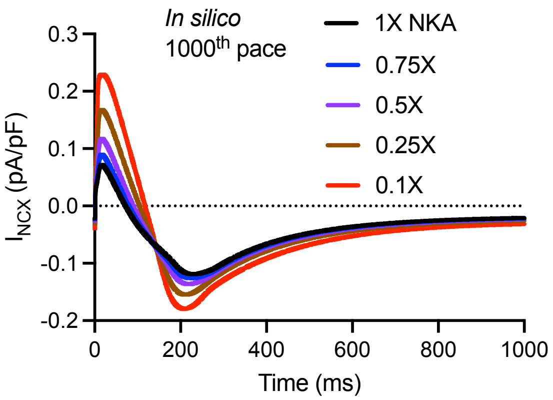

Supplemental Figure 15: Simulated I_{NCX} in the ToR-ORd model of human ventricular cardiomyocyte. Graph illustrates NCX current vs time on the 1000th pace in a set of simulations with different NKA functional states.

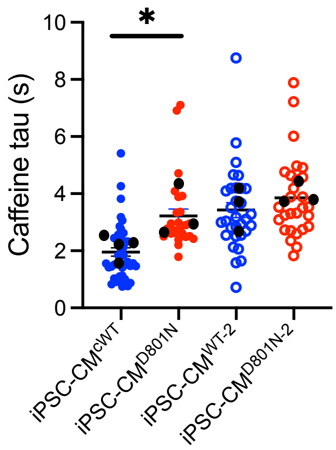

Supplemental Figure 16: Tau of SR store amplitude. Graph shows fluorescence decay constant of each cell after application of caffeine. n= 43, 27, 31, and 29, respectively. Black dots represent experimental means. A hierarchical statistical test was conducted. * $p<0.05$.

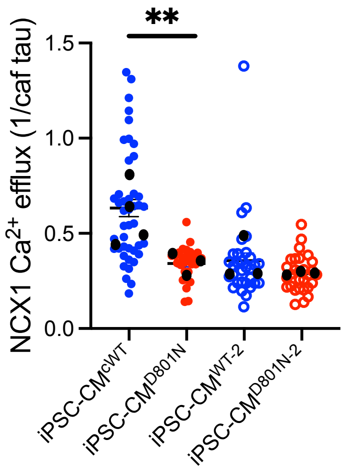

Supplemental Figure 17: Calculated NCX1 activity. Graph illustrates calculated NCX Ca^{2+} efflux for each cell. n= 43, 27, 31, and 29, respectively. Black dots represent experimental means. A hierarchical statistical test was conducted. ** p<0.01.

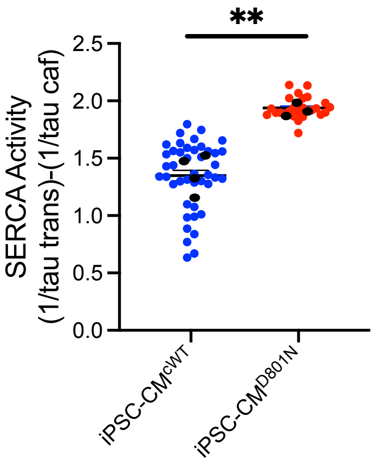

Supplemental Figure 18: Calculated SERCA activity. Graph shows SERCA activity for each cell in iPSC-CM^{cWT} and iPSC-CM^{D801N}. n= 43, 27, respectively. Black dots indicate experimental means. A hierarchical statistical test was conducted, ** $p<0.01$.

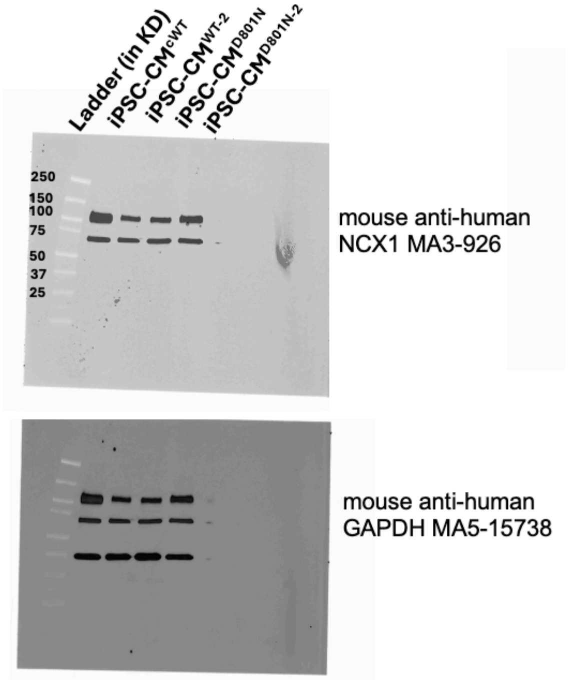

Supplemental Figure 19: Western blot comparing NCX1 expression. Representatives of western blot obtained from whole protein lysates from iPSC-CMs blotting for NCX1 and GAPDH as loading control.

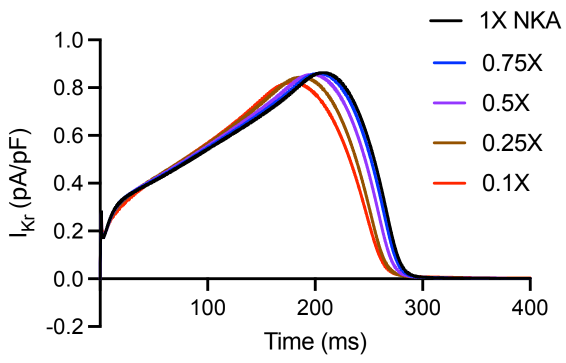

Supplemental Figure 20: Simulated I_{Kr} in the ToR-ORd model of human ventricular cardiomyocyte. Graph illustrates I_{Kr} current vs time on the 1000th pace in a set of simulations with different NKA functional states.

SUPPLEMENTAL REFERENCES

1. Tomek J, Bueno-Orovio A, Passini E, Zhou X, Minchole A, Britton O, et al. Development, calibration, and validation of a novel human ventricular myocyte model in health, disease, and drug block. *Elife.* 2019;8.
2. Moya-Mendez ME, Bidzimou MT, Muralidharan P, Zhang Z, Ezekian JE, Perelli RM, et al. ATP1A3 Variants, Variably Penetrant Short QT Intervals, and Lethal Ventricular Arrhythmias. *JAMA Pediatr.* 2025.
3. Nguyen PT, Deisl C, Fine M, Tippetts TS, Uchikawa E, Bai XC, et al. Structural basis for gating mechanism of the human sodium-potassium pump. *Nat Commun.* 2022;13(1):5293.
4. Sun B, Rouzbehani OMT, Kramer RJ, Ghosh R, Perelli RM, Atkins S, et al. Nonsense Variant PRDM16-Q187X Causes Impaired Myocardial Development and TGF-beta Signaling Resulting in Noncompaction Cardiomyopathy in Humans and Mice. *Circ Heart Fail.* 2023;16(12):e010351.
5. Mary E. Moya-Mendez MD MS MHS1 M-TBB, Padmapriya Muralidharan PhD1, Zhushan Zhang, MD PhD1, Zhushan Zhang, MD PhD1, Priya***, Jordan E. Ezekian MD MPH1,2, Robin M. Perelli MS3, Lauren E. Parker BS1, Lyndsey Prange PNP4, April Boggs RN4, Jeffrey J. Kim MD5, Taylor S. Howard MD5, Tarah A. Word, PhD6,7, Xander H. T. Wehrens MD PhD5,6,7, Gabriela Reyes Valenzuela MD8, Roberto Caraballo MD8, Giacomo Garone MD9, Federico Vigevano MD9, Sarah Weckhuysen MD PhD10.11,12, Charissa Millevert MD10,11, Monica Troncoso MD13, Mario Matamala MD13, Simona Balestrini MD PhD14,15, Sanjay M. Sisodiya PhD FRCP14, Josephine Poole MSc14, Claudio Zucca MD16, Eleni Panagiotakaki MD PhD17, Maria T. Papadopoulou MD17, Sébile Tchaicha MS17, Julie Sauquet LLM17, Marta Zawadzka MD PhD18, Maria Mazurkiewicz-Bełdzińska MD18, Carmen Fons MD PhD19, Jennifer Anticona MD19, Elisa De Grandis MD20,21, Ramona Cordani MD20,21, Livia Pisciotta MD, PhD22, Sergiu Groppa MD23, Sandra Paryjas MD23, Francesca Ragona MD24, Elena Mangia RN24, Tiziana Granata MD24, Andrey Megvinov PhD25, Mirjana Pavlicek MD26, Kevin Ess MD, PhD27, Christine Q. Simmons MD28, Alfred L George, Jr., MD28, Rosaria Vavassori PhD25,296,3027, Mohamad A. Mikati MD4#, Andrew P. Landstrom MD PhD1. ATP1A3 Variants are Associated with Variably Penetrant Short QT and Lethal Ventricular Arrhythmias *JAMA Pediatr.* 2024;Accepted.
6. Burridge PW, Matsa E, Shukla P, Lin ZC, Churko JM, Ebert AD, et al. Chemically defined generation of human cardiomyocytes. *Nat Methods.* 2014;11(8):855-60.
7. de Carvalho Aguiar P, Sweadner KJ, Penniston JT, Zaremba J, Liu L, Caton M, et al. Mutations in the Na+/K+ -ATPase alpha3 gene ATP1A3 are associated with rapid-onset dystonia parkinsonism. *Neuron.* 2004;43(2):169-75.
8. Shinnawi R, Huber I, Maizels L, Shaheen N, Gepstein A, Arbel G, et al. Monitoring Human-Induced Pluripotent Stem Cell-Derived Cardiomyocytes with Genetically Encoded Calcium and Voltage Fluorescent Reporters. *Stem Cell Reports.* 2015;5(4):582-96.

9. Tomek J, Nieves-Cintron M, Navedo MF, Ko CY, and Bers DM. SparkMaster 2: A New Software for Automatic Analysis of Calcium Spark Data. *Circ Res.* 2023;133(6):450-62.
10. Voigt N, Heijman J, Wang Q, Chiang DY, Li N, Karck M, et al. Cellular and molecular mechanisms of atrial arrhythmogenesis in patients with paroxysmal atrial fibrillation. *Circulation.* 2014;129(2):145-56.
11. Alsina KM, Hulsurkar M, Brandenburg S, Kownatzki-Danger D, Lenz C, Urlaub H, et al. Loss of Protein Phosphatase 1 Regulatory Subunit PPP1R3A Promotes Atrial Fibrillation. *Circulation.* 2019;140(8):681-93.
12. Ma J, Guo L, Fiene SJ, Anson BD, Thomson JA, Kamp TJ, et al. High purity human-induced pluripotent stem cell-derived cardiomyocytes: electrophysiological properties of action potentials and ionic currents. *Am J Physiol Heart Circ Physiol.* 2011;301(5):H2006-17.
13. Guo F, Sun Y, Wang X, Wang H, Wang J, Gong T, et al. Patient-Specific and Gene-Corrected Induced Pluripotent Stem Cell-Derived Cardiomyocytes Elucidate Single-Cell Phenotype of Short QT Syndrome. *Circ Res.* 2019;124(1):66-78.
14. Wu Y, Valdivia HH, Wehrens XH, and Anderson ME. A Single Protein Kinase A or Calmodulin Kinase II Site Does Not Control the Cardiac Pacemaker Ca2+ Clock. *Circ Arrhythm Electrophysiol.* 2016;9(2):e003180.
15. Perelli RM, Dewars ER, Cope H, Behura AS, Ponek AQ, Sala AM, et al. TAX1BP3 Causes TRPV4-Mediated Autosomal Recessive Arrhythmogenic Cardiomyopathy. *Circ Res.* 2025;136(7):667-84.
16. Zhang ZS, Cheng HJ, Ukai T, Tachibana H, and Cheng CP. Enhanced cardiac L-type calcium current response to beta2-adrenergic stimulation in heart failure. *J Pharmacol Exp Ther.* 2001;298(1):188-96.
17. Seibertz F, Rapedius M, Fakuade FE, Tomsits P, Liutkute A, Cyganek L, et al. A modern automated patch-clamp approach for high throughput electrophysiology recordings in native cardiomyocytes. *Commun Biol.* 2022;5(1):969.
18. Sikkel MB, Francis DP, Howard J, Gordon F, Rowlands C, Peters NS, et al. Hierarchical statistical techniques are necessary to draw reliable conclusions from analysis of isolated cardiomyocyte studies. *Cardiovasc Res.* 2017;113(14):1743-52.
19. Jewell EA, and Lingrel JB. Comparison of the substrate dependence properties of the rat Na,K-ATPase alpha 1, alpha 2, and alpha 3 isoforms expressed in HeLa cells. *J Biol Chem.* 1991;266(25):16925-30.
20. Ruan DD, Zou J, Liao LS, Ji MD, Wang RL, Zhang JH, et al. In vitro study of ATP1A3 p.Ala275Pro mutant causing alternating hemiplegia of childhood and rapid-onset dystonia-parkinsonism. *Front Neurosci.* 2024;18:1415576.
21. Depierre P, Ginet V, Truttmann AC, and Puyal J. Neuronal autosis is Na(+)/K(+)-ATPase alpha 3-dependent and involved in hypoxic-ischemic neuronal death. *Cell Death Dis.* 2024;15(5):363.
22. Kim W, Underwood RS, Greenwald I, and Shaye DD. OrthoList 2: A New Comparative Genomic Analysis of Human and Caenorhabditis elegans Genes. *Genetics.* 2018;210(2):445-61.

23. Ng HWY, Ogbeta JA, and Clapcote SJ. Genetically altered animal models for ATP1A3-related disorders. *Dis Model Mech.* 2021;14(10).
