# Supplementary material for: D801N in ATP1A3-encoded Na/K-ATPase alpha 3 causes cardiac arrhythmogenesis through sodium-calcium exchanger–mediated calcium overload
Source: JCI Insight. 2026 Apr 8;11(7):e197721. doi: 10.1172/jci.insight.197721 (PMC13134723; doi:10.1172/jci.insight.197721)
Supplement: Unedited blot and gel images [file jciinsight-11-197721-s087.pdf]

D801N in ATP1A3-encoded Na/K-ATPase Alpha 3 causes Cardiac Arrhythmogenesis through Sodium-Calcium Exchanger-mediated Calcium Overload

Unedited western blot images

Figure 1B

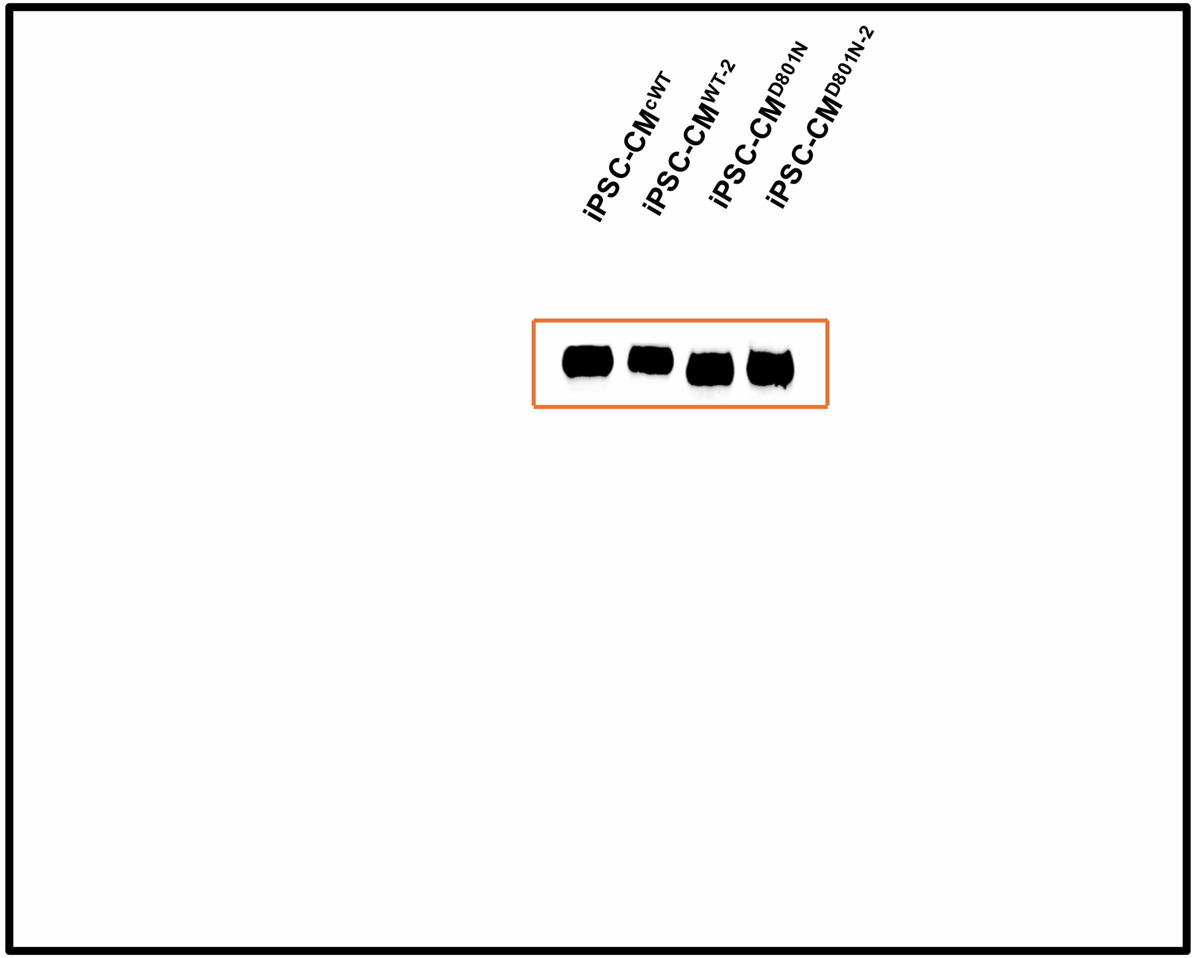

chemiluminescence

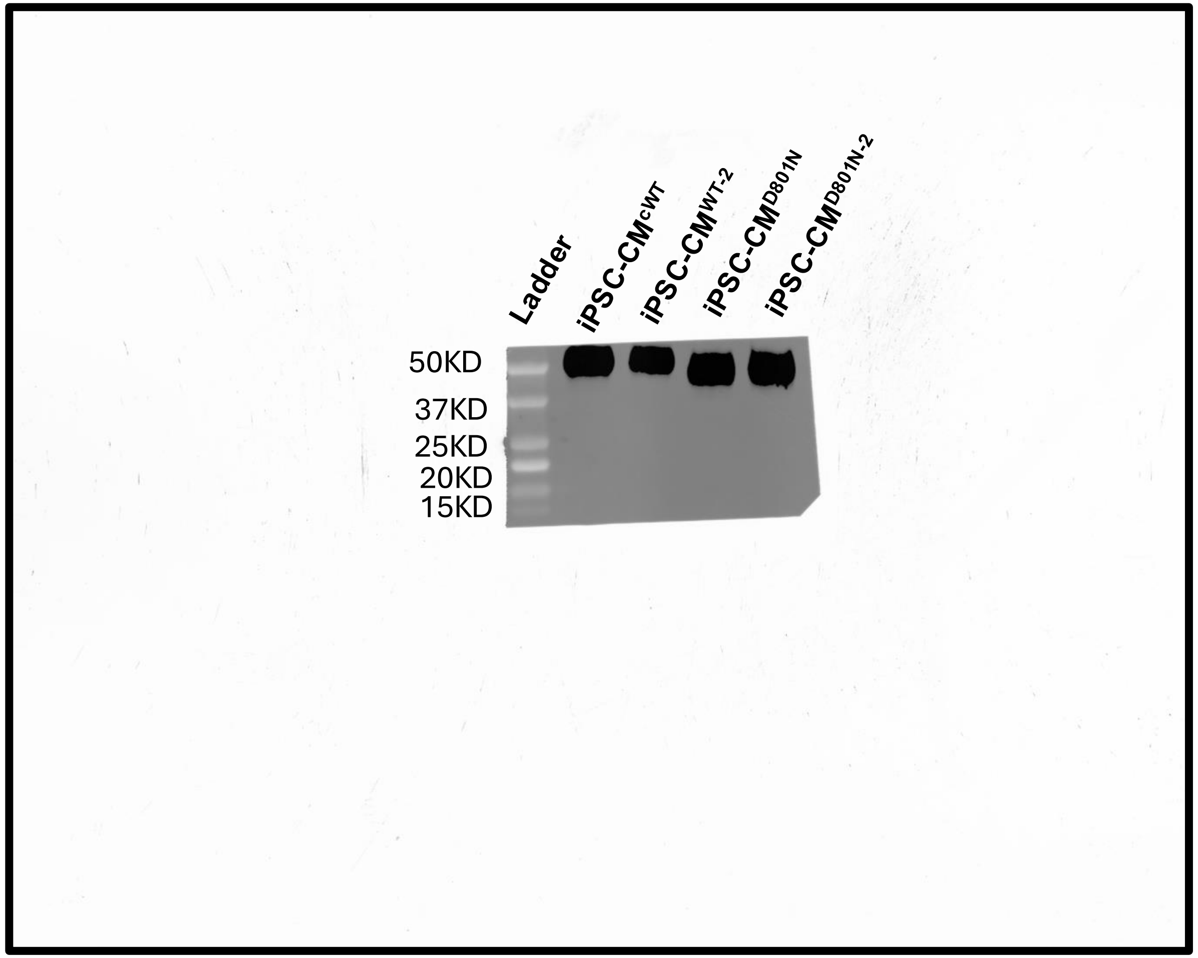

chemiluminescence + colorimetric (for ladder)

Unedited images of Western blots for Figure 1B. Protein lysates for iPSC-CMs were blotted with mouse anti-human ATP1B1 (cat# MA3930, ThermoFisher). Highlighted box indicates cropped image used in main figure

Figure 1B

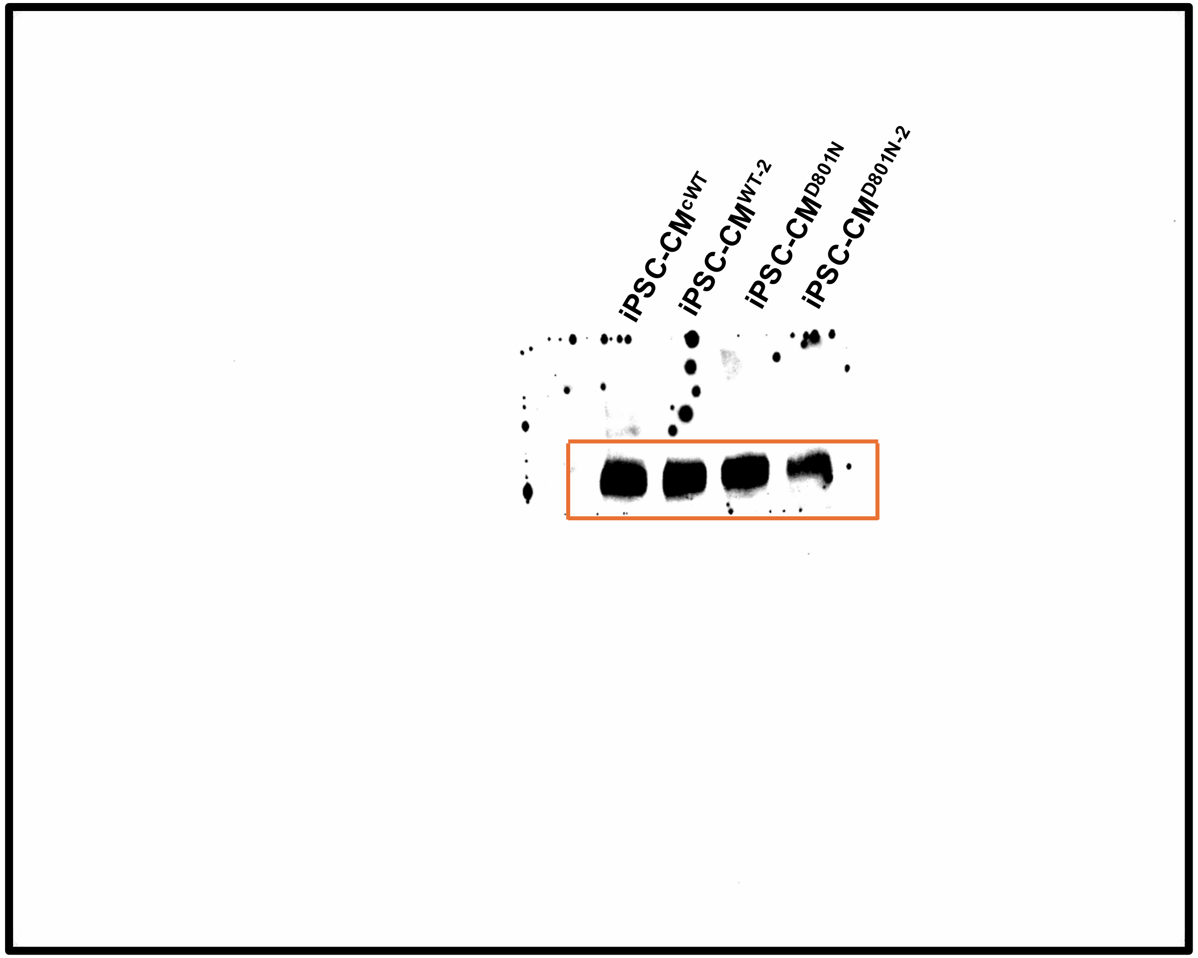

chemiluminescence

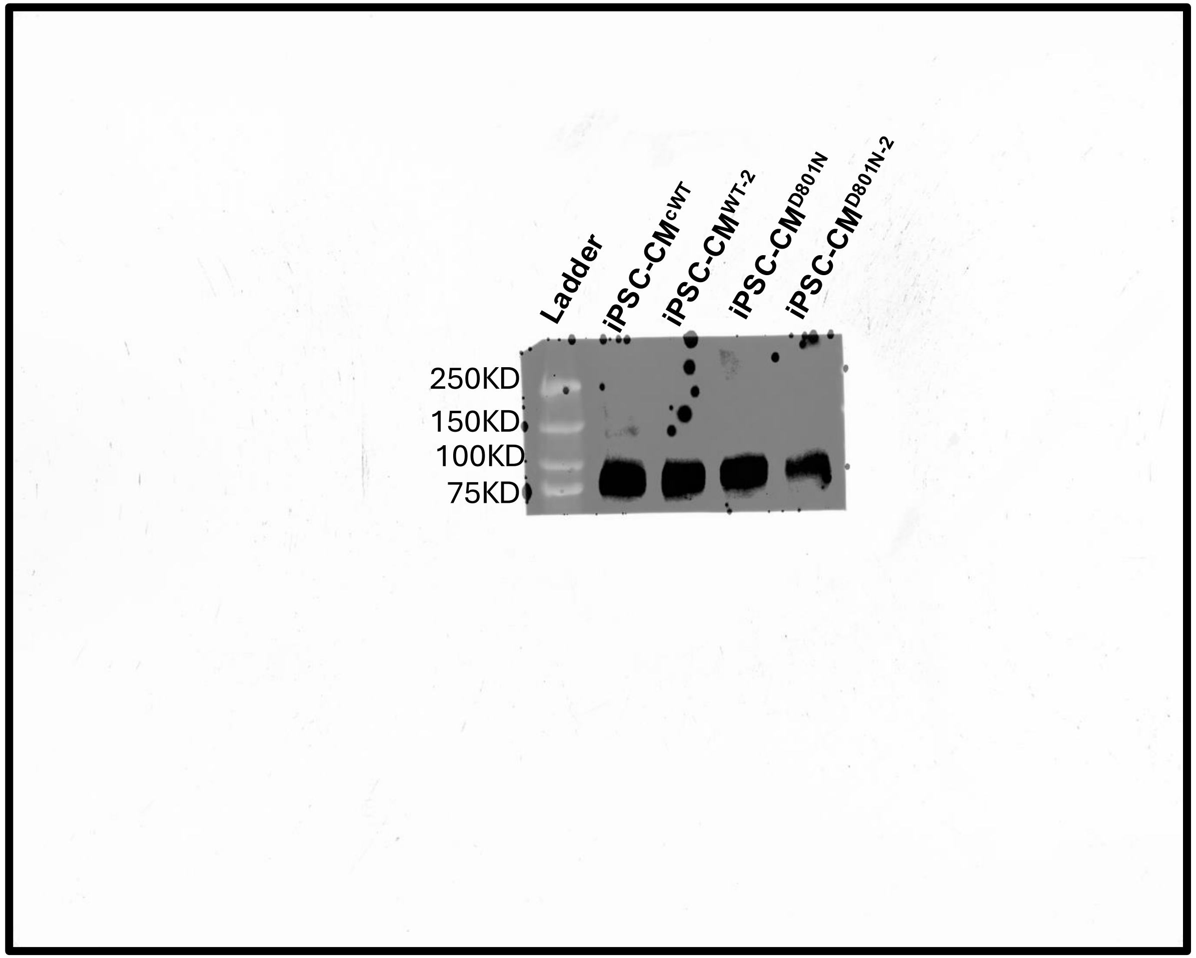

chemiluminescence + colorimetric (for ladder)

Unedited images of Western blots for Figure 1B. Protein lysates for iPSC-CMs were blotted with mouse anti-human ATP1A1 (cat# ab7671, Abcam). Highlighted box indicates cropped image used in main figure

Figure 1B

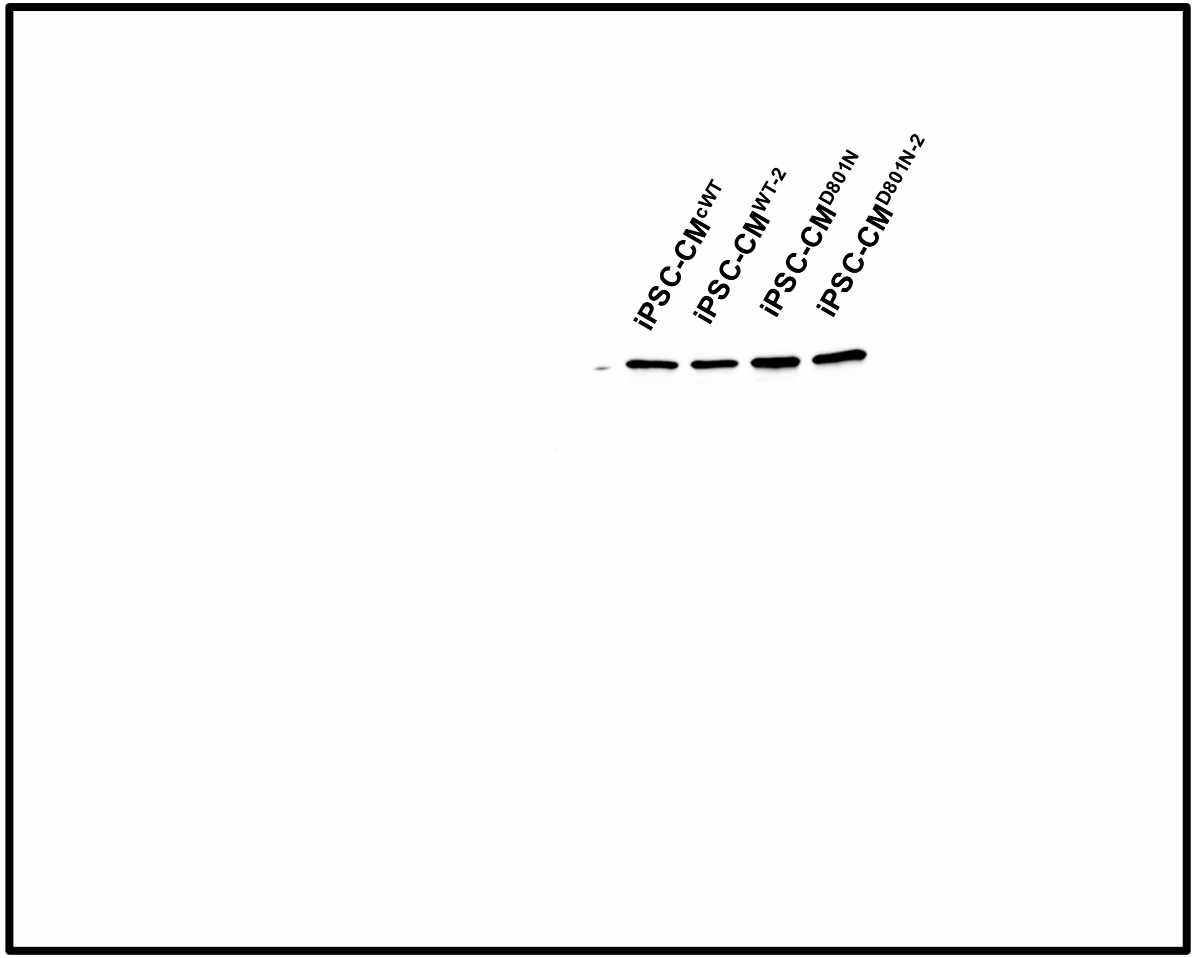

chemiluminescence

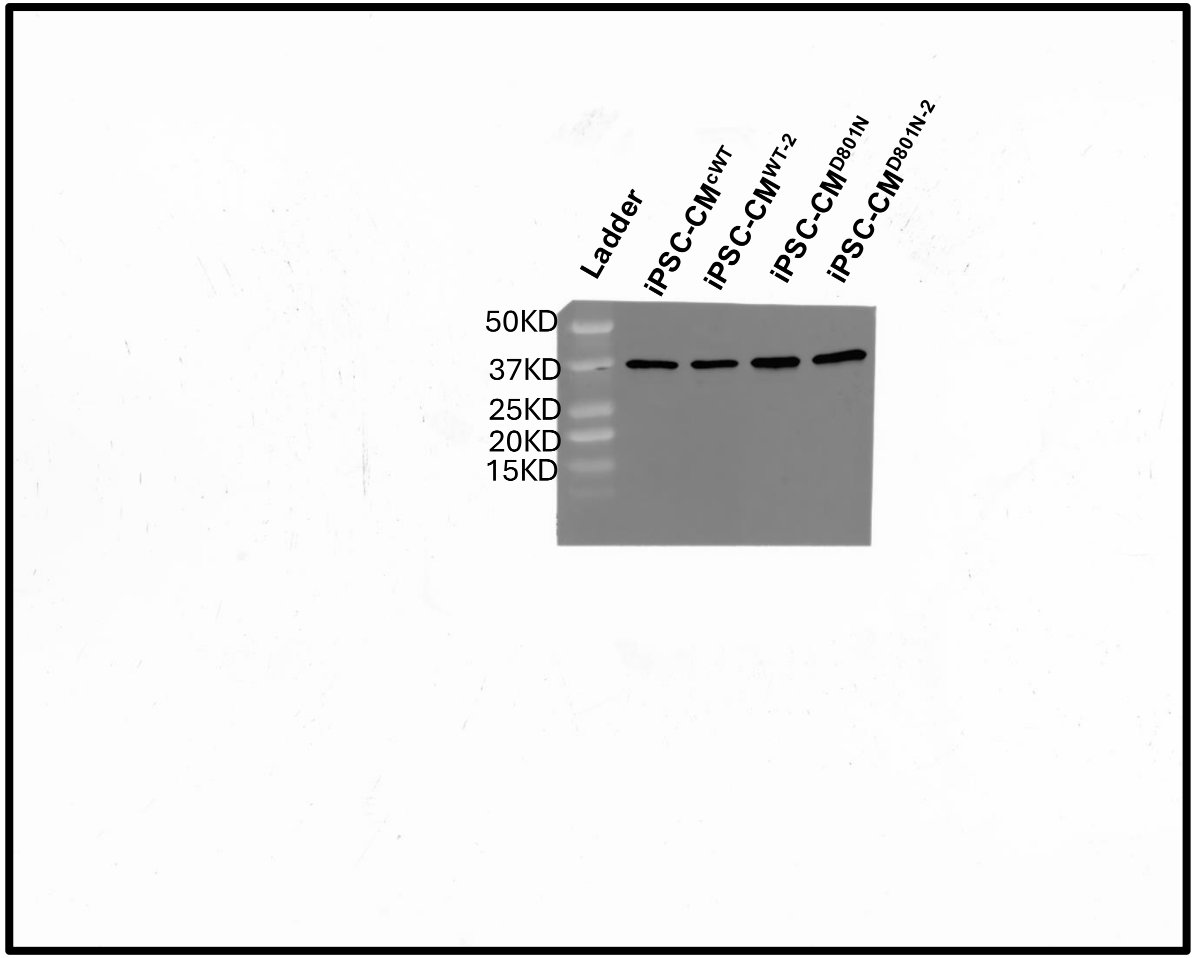

chemiluminescence + colorimetric (for ladder)

Unedited images of Western blots for Figure 1B. Protein lysates for iPSC-CMs were blotted with mouse anti-human GAPDH (cat# MA5-15738, ThermoFisher). This blot serves as loading control for corresponding ATP1A1 Western blot.

Figure 1B

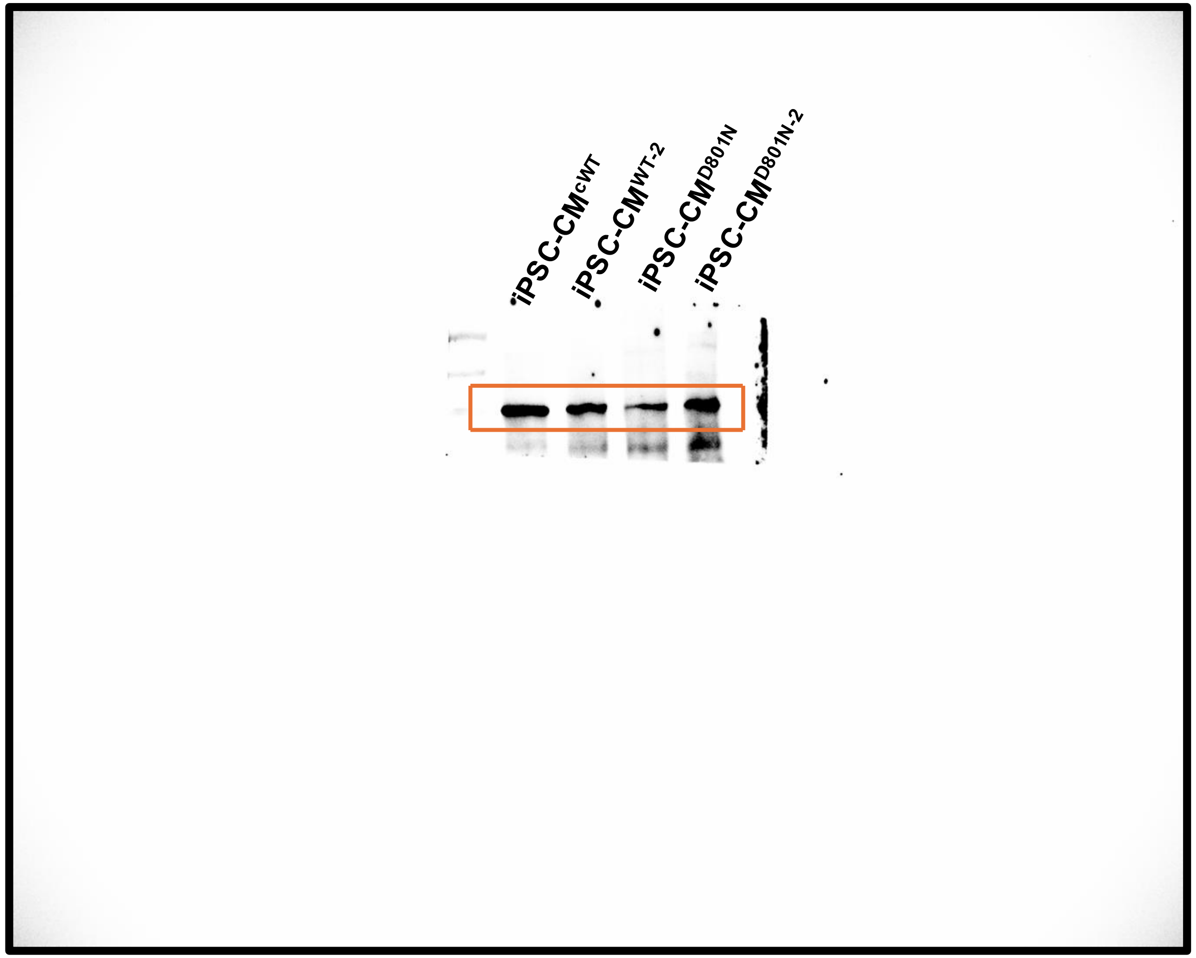

chemiluminescence

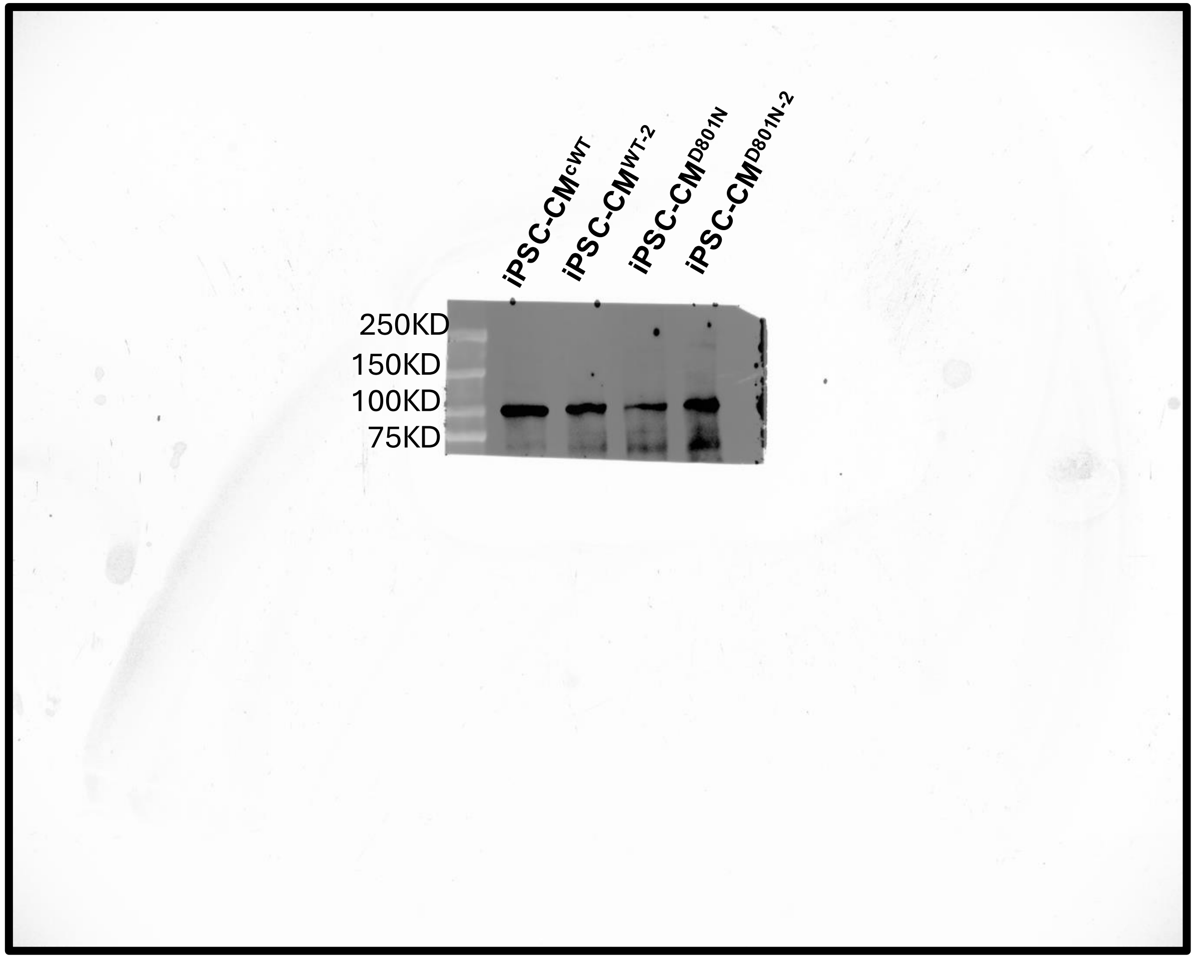

chemiluminescence + colorimetric (for ladder)

Unedited images of Western blots for Figure 1B. Protein lysates for iPSC-CMs were blotted with rabbit anti-human ATP1A2 (cat# ab166888, Abcam). Highlighted box indicates cropped image used in main figure.

Figure 1B

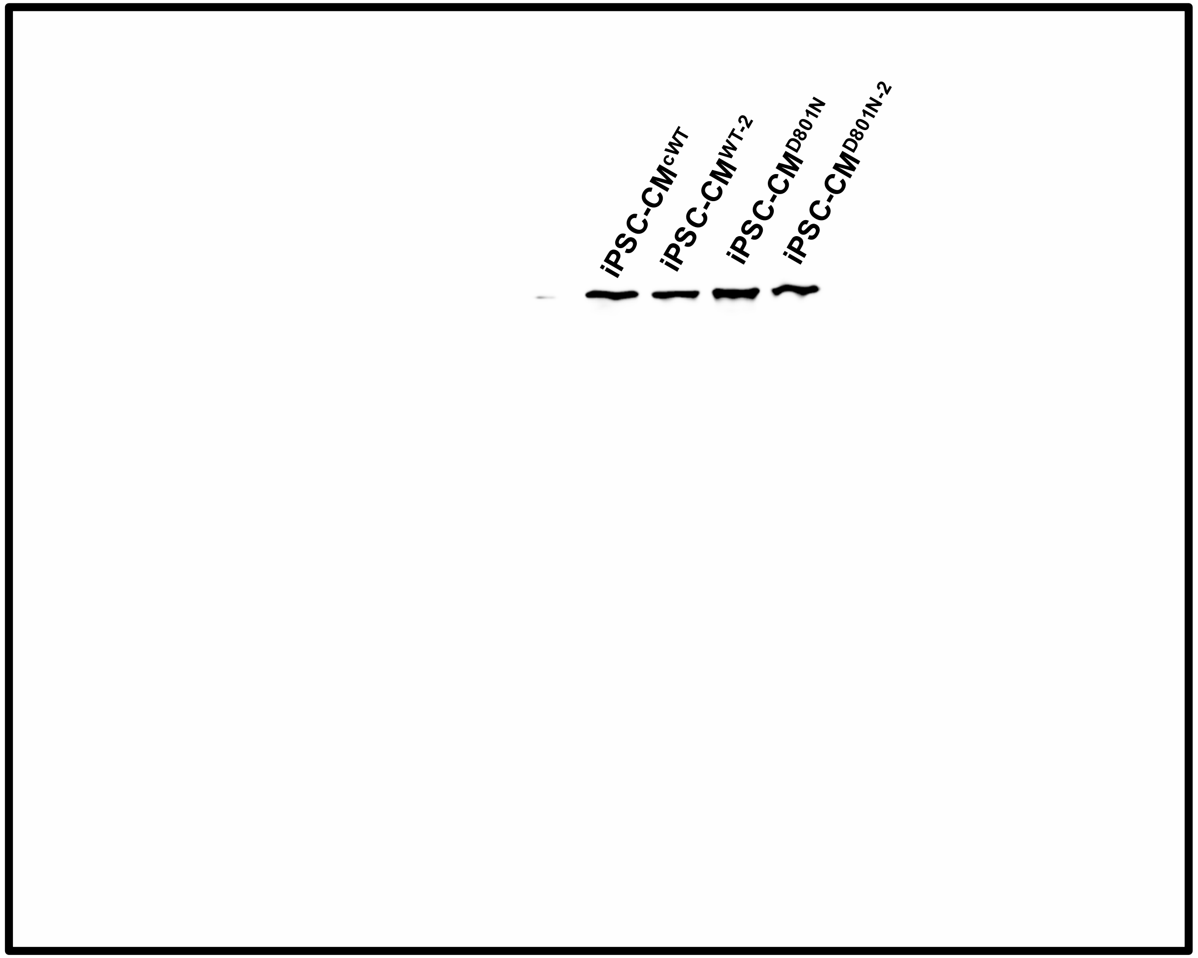

chemiluminescence

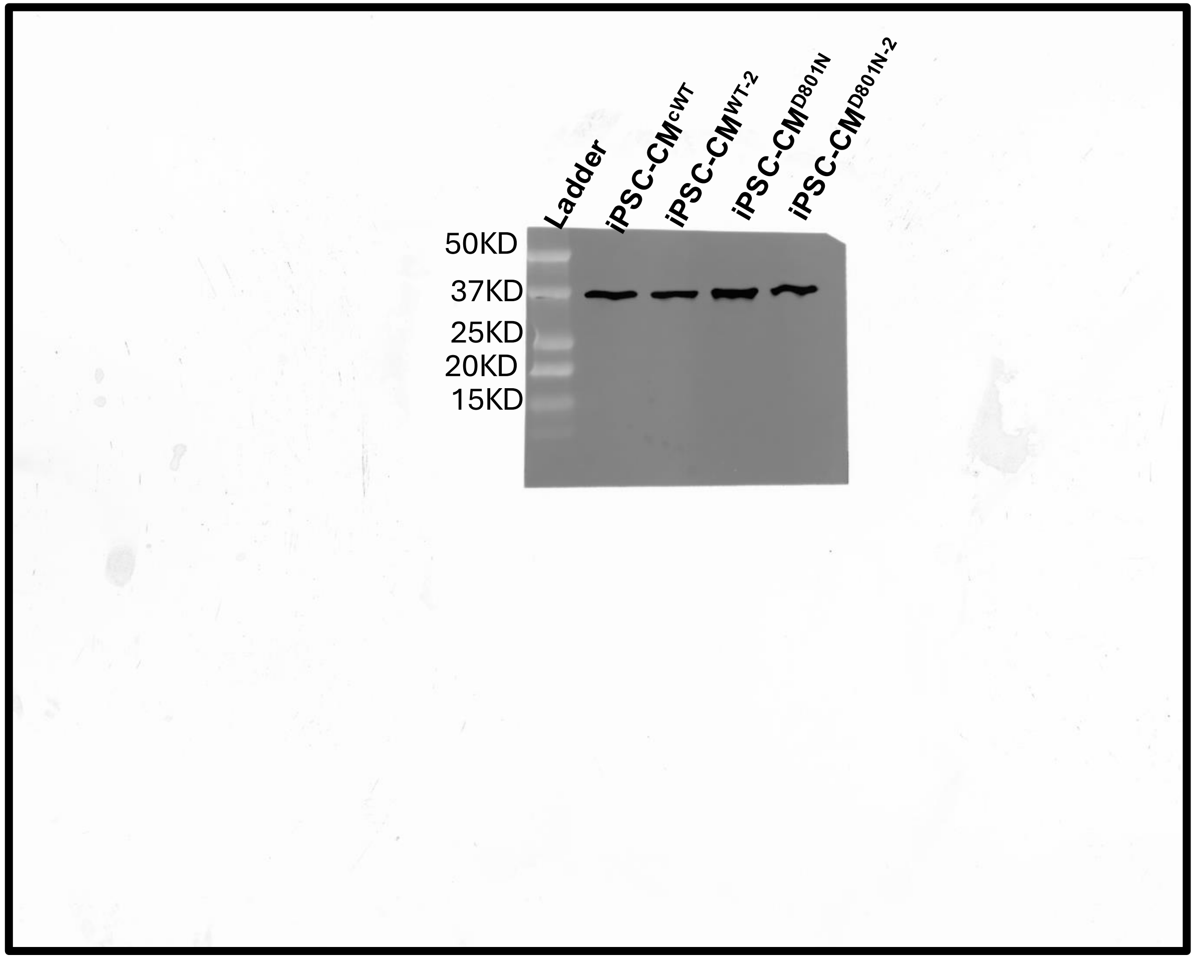

chemiluminescence + colorimetric (for ladder)

Unedited images of Western blots for Figure 1B. Protein lysates for iPSC-CMs were blotted with mouse anti-human GAPDH (cat# MA5-15738, ThermoFisher). This blot was used as loading control for corresponding ATP1A2 Western Blot.

Figure 1B

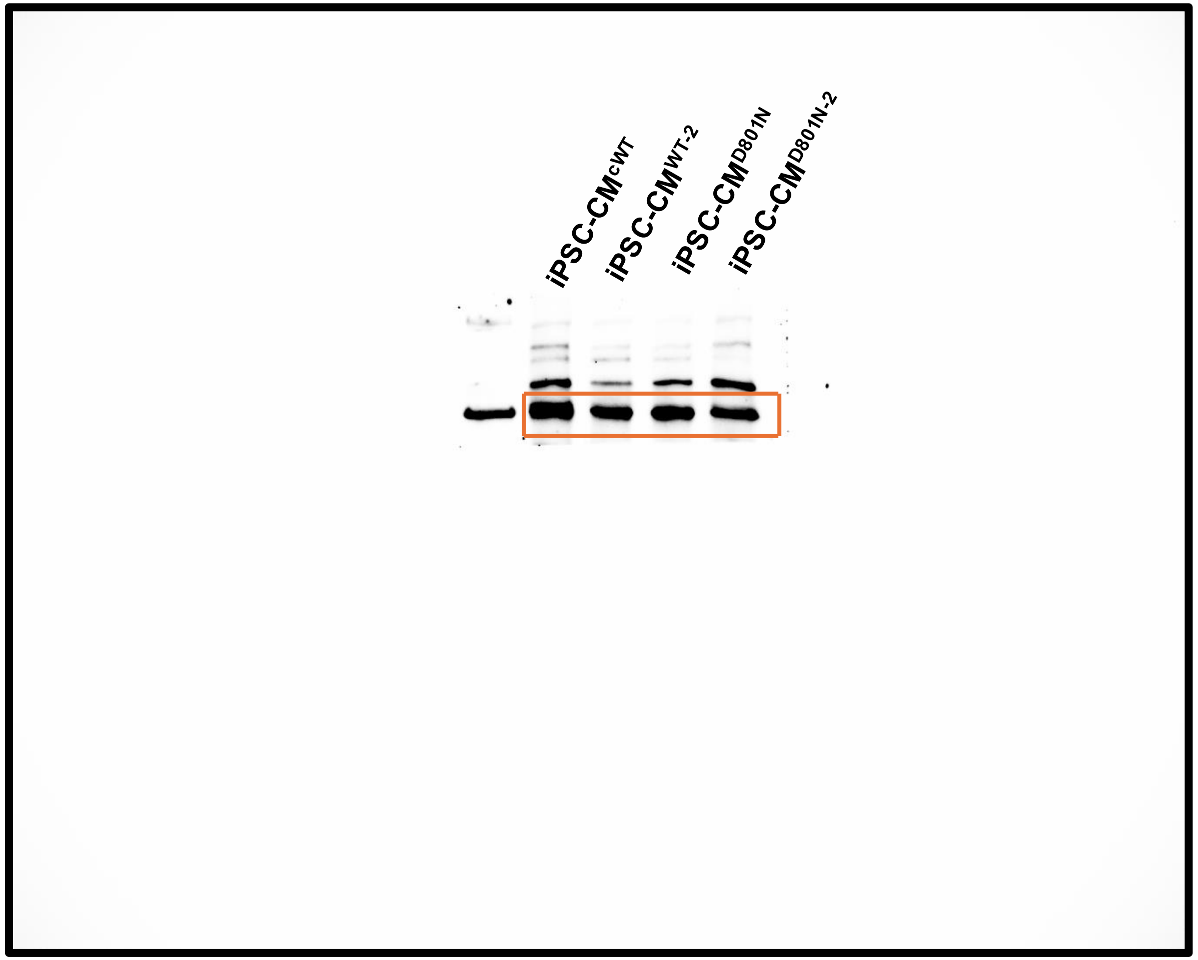

chemiluminescence

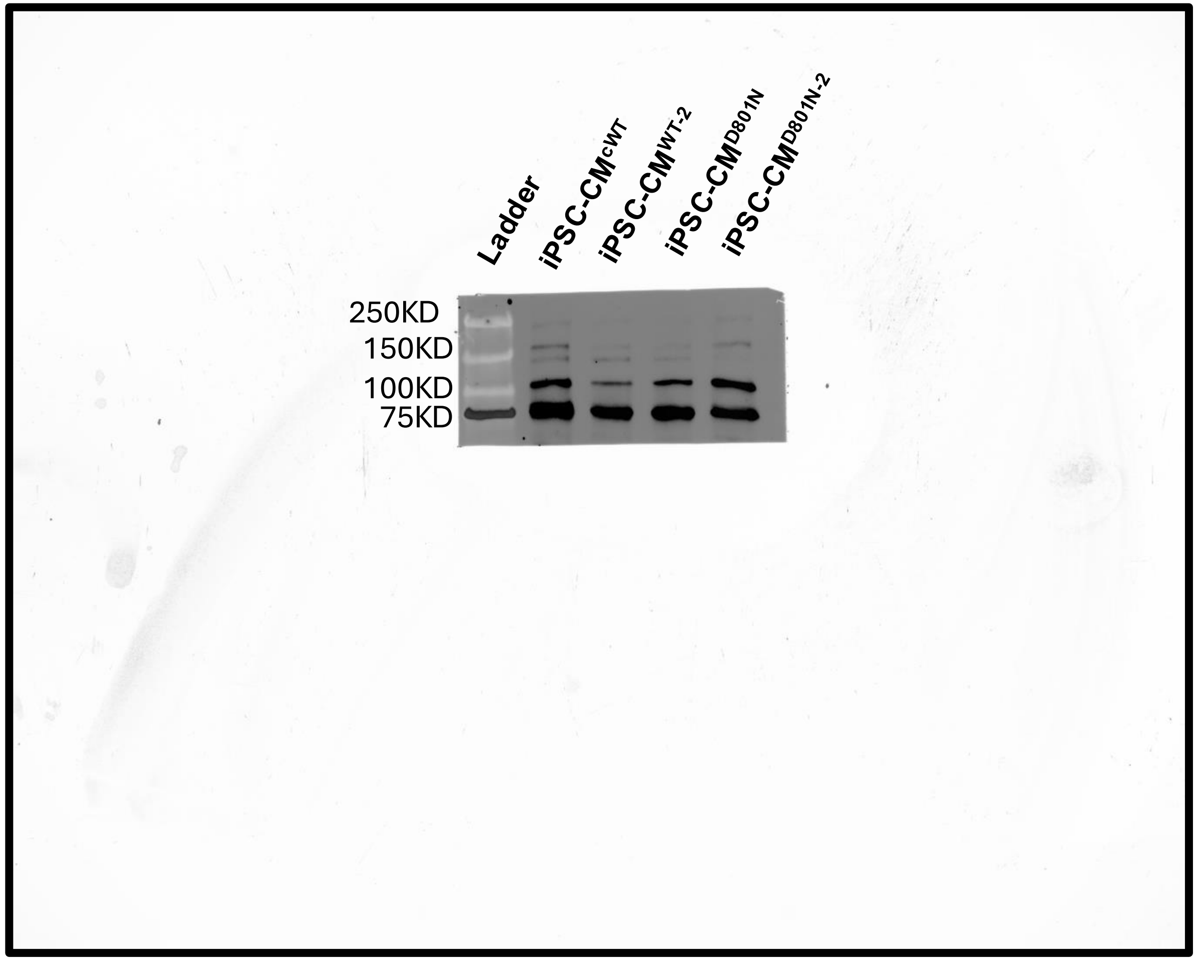

chemiluminescence + colorimetric (for ladder)

Unedited images of Western blots for Figure 1B. Protein lysates for iPSC-CMs were blotted with rabbit anti-human ATP1A3 (cat# 06-172-I, Millipore Sigma). Highlighted box indicates cropped image used in main figure.

Figure 1B

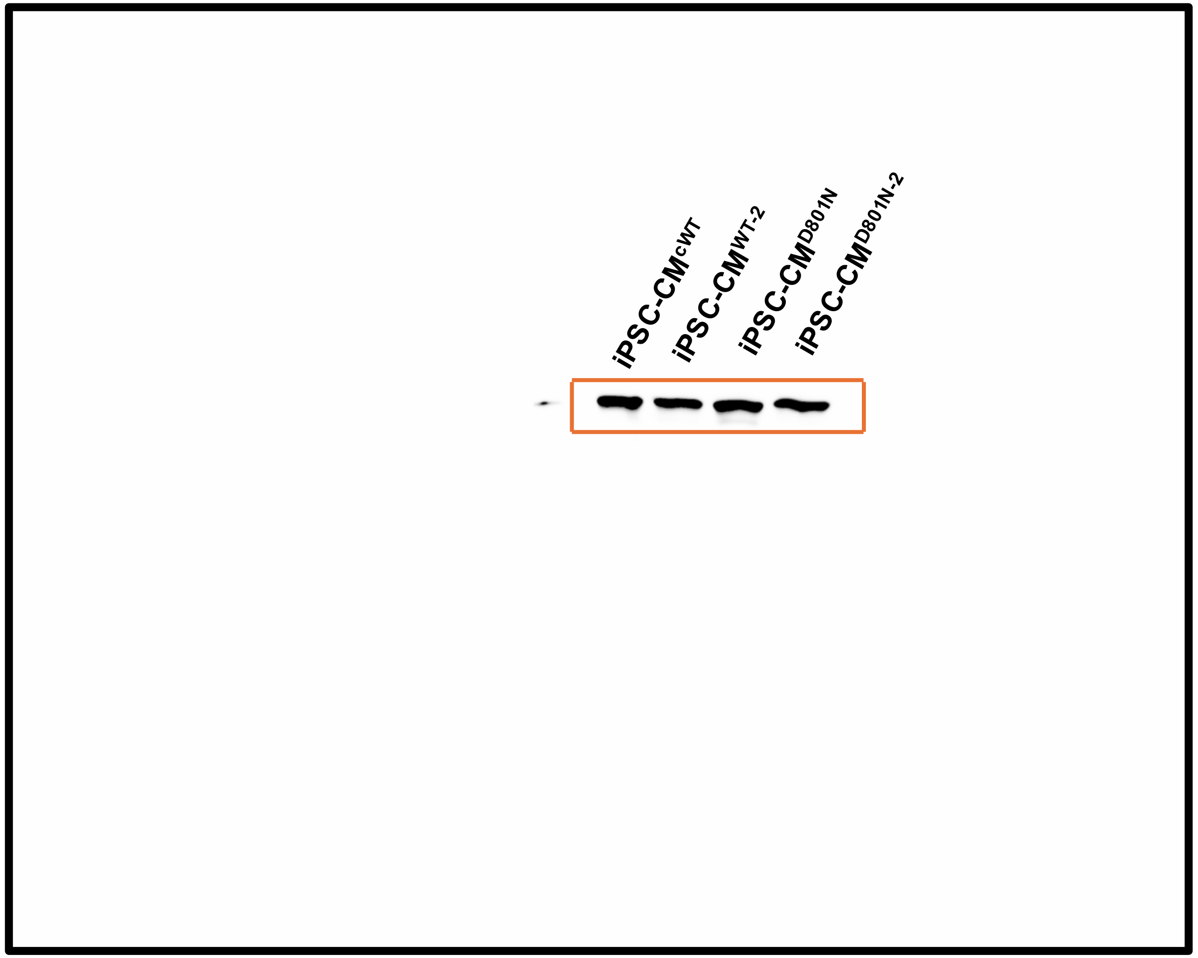

chemiluminescence

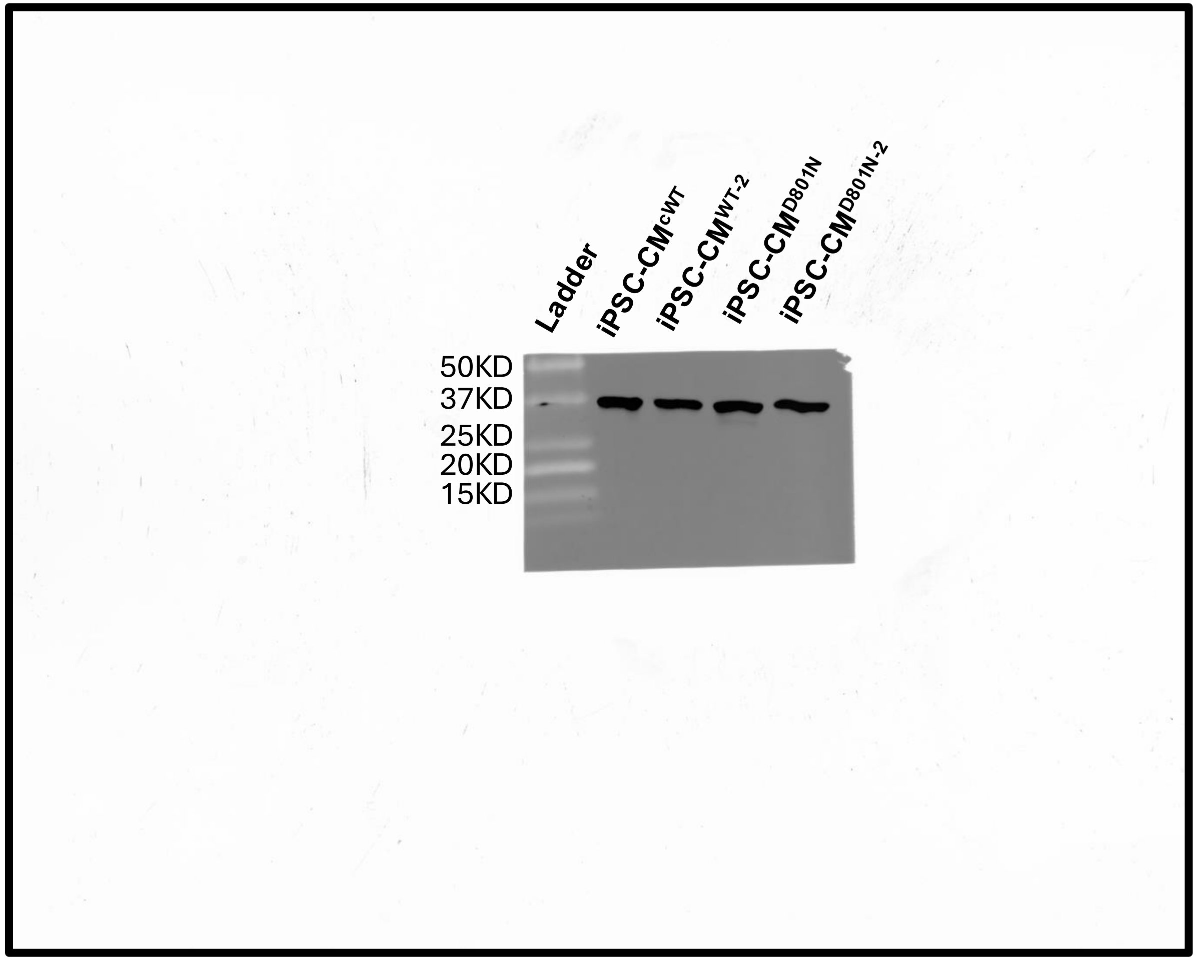

chemiluminescence + colorimetric (for ladder)

Unedited images of Western blots for Figure 1B. Protein lysates for iPSC-CMs were blotted with mouse anti-human GAPDH (cat# MA5-15738, ThermoFisher). This was used as a loading control for corresponding ATP1A3 Western blot. Highlighted box indicates cropped image used in main figure.

Supplemental Figure 18

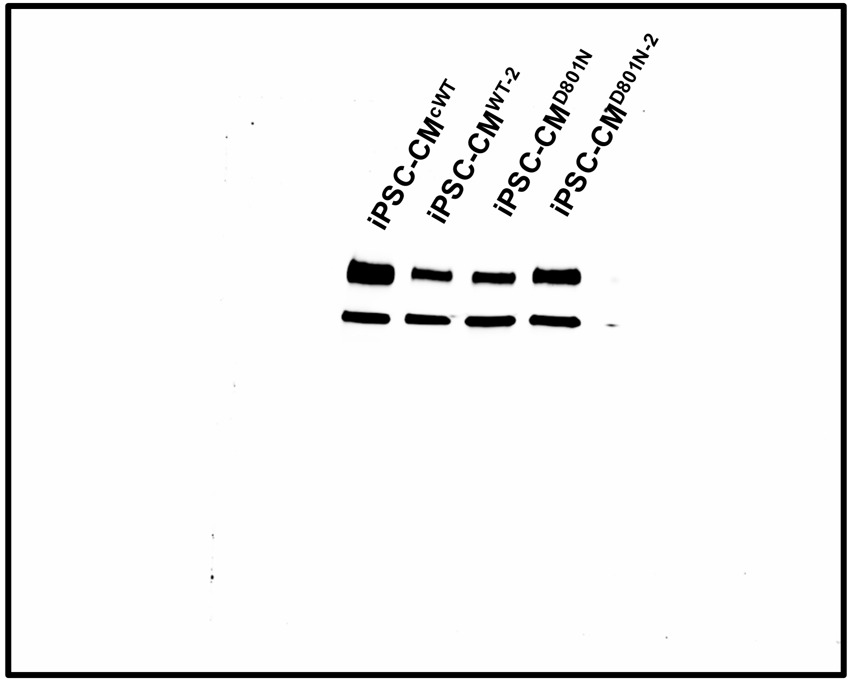

chemiluminescence

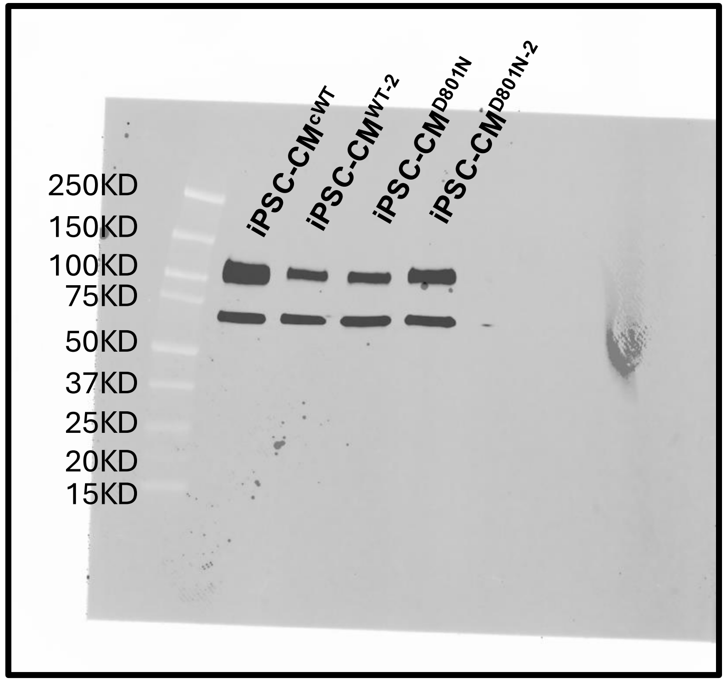

chemiluminescence + colorimetric (for ladder)

Unedited images of Western blots for Supplemental Figure 18. Protein lysates for iPSC-CMs were blotted with mouse anti-human NCX1 (cat# MA3-926, ThermoFisher)

Supplemental Figure 18

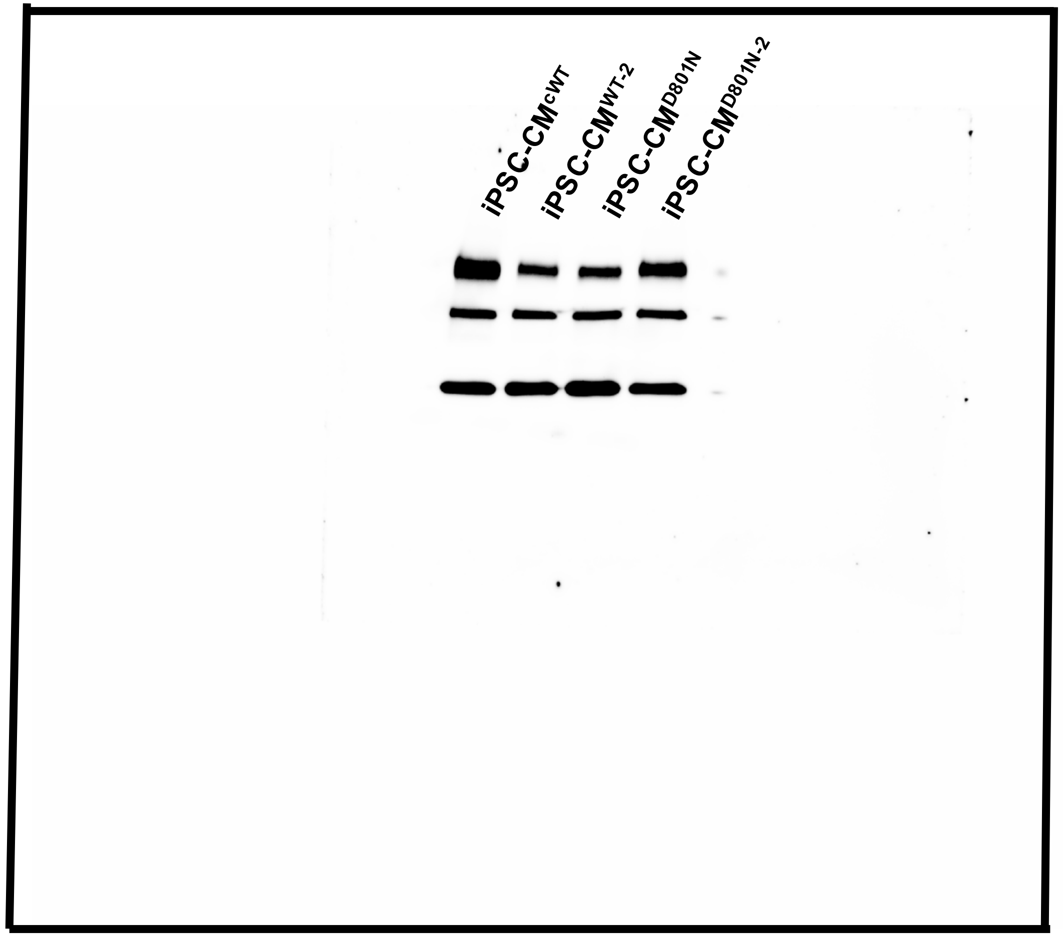

chemiluminescence

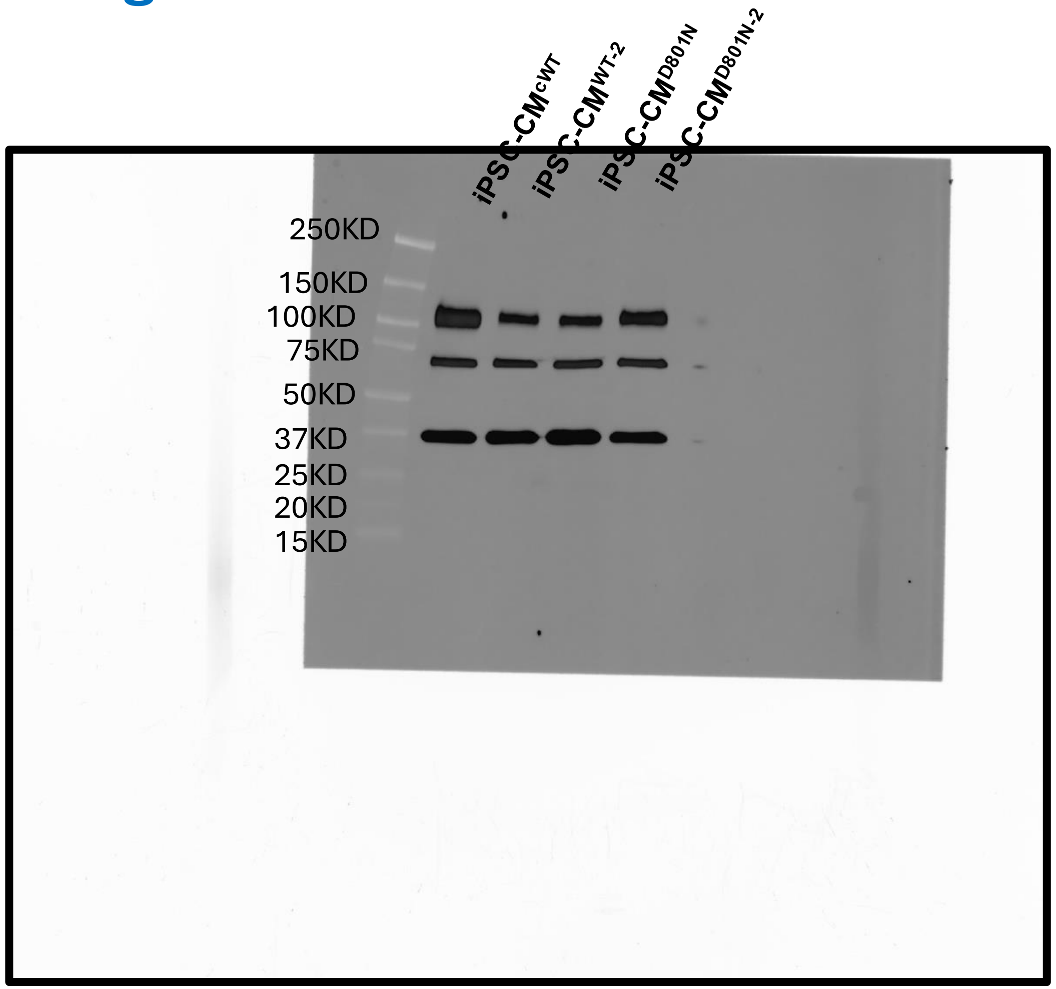

chemiluminescence + colorimetric (for ladder)

Unedited images of Western blots for Supplemental Figure 18. Protein lysates for iPSC-CMs were blotted with mouse anti-human GAPDH (cat# MA5-15738, ThermoFisher)
